# Automating expert-level medical reasoning evaluation of large language models

**DOI:** 10.1038/s41746-025-02208-7

**Published:** 2025-12-06

**Authors:** Shuang Zhou, Wenya Xie, Jiaxi Li, Zaifu Zhan, Meijia Song, Han Yang, Cheyenna Espinoza, Lindsay Welton, Xinnie Mai, Yanwei Jin, Zidu Xu, Yuen-Hei Chung, Yiyun Xing, Meng-Han Tsai, Emma Schaffer, Yucheng Shi, Ninghao Liu, Zirui Liu, Rui Zhang

**Affiliations:** 1 Division of Computational Health Sciences, Department of Surgery, University of Minnesota, Minneapolis, MN USA; 2 College of Science and Engineering, University of Minnesota, Minneapolis, MN USA; 3 School of Computing, University of Georgia, Athens, GA USA; 4 Department of Electrical and Computer Engineering, University of Minnesota, Minneapolis, MN USA; 5 School of Nursing, University of Minnesota, Minneapolis, MN USA; 6 Institute for Health Informatics, University of Minnesota, Minneapolis, MN USA; 7 Department of Surgery, University of Minnesota, Minneapolis, MN USA; 8 School of Data Science, University of Virginia, Charlottesville, VA USA; 9 Division of Biostatistics & Health Data Science, University of Minnesota, Minneapolis, MN USA; 10 School of Nursing, Columbia University, New York, NY USA; 11 Division of Cardiac Electrophysiology, University of California, San Francisco, San Francisco, CA USA; 12 School of Dentistry, University of Minnesota, Minneapolis, MN USA; 13 Division of Cardiothoracic Surgery, Department of Surgery, University of Colorado Anschutz Medical Campus, Aurora, CO USA

**Keywords:** Business and industry, Computational biology and bioinformatics, Health care, Mathematics and computing, Medical research

## Abstract

As large language models (LLMs) become increasingly integrated into clinical decision-making, ensuring trustworthy reasoning is paramount. However, current evaluation strategies of LLMs’ medical reasoning capability either suffer from unsatisfactory assessment or poor scalability, and a rigorous benchmark remains absent. To address this, we present MedThink-Bench, a benchmark designed for rigorous and scalable assessment of LLMs’ medical reasoning. MedThink-Bench comprises 500 high-complexity questions spanning ten medical domains, accompanied by expert-authored, step-by-step rationales that elucidate intermediate reasoning processes. Further, we introduce LLM-w-Rationale, an evaluation framework that combines fine-grained rationale assessment with an LLM-as-a-Judge paradigm, enabling expert-level fidelity in evaluating reasoning quality while preserving scalability. Results show that LLM-w-Rationale correlates strongly with expert evaluation (Pearson coefficient up to 0.87) while requiring only 1.4% of the evaluation time. Overall, MedThink-Bench establishes a rigorous and scalable standard for evaluating medical reasoning in LLMs, advancing their safe and responsible deployment in clinical practice.

## Introduction

Large language models (LLMs) have made remarkable progress in clinical decision-making, demonstrating the ability to perform complex reasoning tasks such as disease diagnosis^1,2^, treatment planning^3^, and patient management^4,5^. Despite their impressive capabilities, the opaque and black-box nature of LLMs limits their reliability in high-stakes clinical scenarios^6,7^. For instance, an LLM may arrive at the correct diagnosis based on parametric knowledge without providing evidence grounded in clinical guidelines or considering a comprehensive differential diagnosis^8–10^. Moreover, LLMs are prone to hallucinations, generating plausible but factually incorrect information that can mislead clinical decision-making^11–13^. Such behavior poses potential risks to patient safety and undermines the reliability of clinical workflows^14^. Therefore, deploying LLMs in clinical practice requires not only high prediction accuracy but also transparent and explainable reasoning processes^15^.

Evaluating the medical reasoning capabilities of LLMs is crucial for establishing trust and ensuring safe integration into healthcare settings^16–18^. Recent efforts in this direction have followed two main approaches. The first approach involves assessing performance on complex medical exercises, such as multiple-choice questions (MCQs), by measuring prediction accuracy^19–21^. While this method aligns with the prevailing approaches to evaluating LLMs’ medical capabilities and offers a coarse measurement^22,23^, it fails to capture the depth and validity of the reasoning process that underpins clinical decisions and cannot identify flawed reasoning^24,25^. The second approach focuses on evaluating the rationales provided by LLMs, which can well address the above issues. Within this category, evaluation strategies can be further classified into three types: (1) text-similarity metrics, which compare LLM-generated rationales with reference rationales^26,27^; (2) human expert evaluation, which relies on domain experts’ manual efforts to assess the reasoning quality^15,25,28,29^; and (3) LLM-as-a-Judge, where a separate LLM is used to assess the quality of the reasoning process^30,31^.

Despite these advances, existing evaluation strategies either suffer from unsatisfactory assessment or poor scalability. Specifically, while conventional text similarity metrics are scalable and cost-efficient^6^, such as those based on lexical-level overlap (e.g., BLUE^32^, ROUGE^33^) or semantic similarity (e.g., BERTScore^34^), they fail to capture medical semantics or nuanced logics and lack robustness to the variance of the text’s expression styles^6^. In contrast, human evaluation remains the gold standard for assessing factuality and nuance but is labor-intensive and limited in scale^25^. Additionally, LLM-as-a-Judge offers a scalable alternative and can comprehend medical knowledge^30,35^, yet it is vulnerable to hallucinations and evaluative bias^36^. As such, the challenge of conducting automated, scalable evaluations of LLMs’ medical reasoning while maintaining expert-level factuality remains unresolved. A further barrier is the lack of a benchmark designed to evaluate LLM-generated medical rationales rigorously. Existing datasets often suffer from narrow clinical scenarios^37^ or rely on LLM-generated rationales as reference answers^31,37,38^, which may involve incorrect knowledge or flawed rationale. This is because the credibility of such artificial intelligence (AI)-generated rationales is uncertain^9,39,40^, and the alignment with human expert judgment remains unclear^41^.

**Fig. 1:**
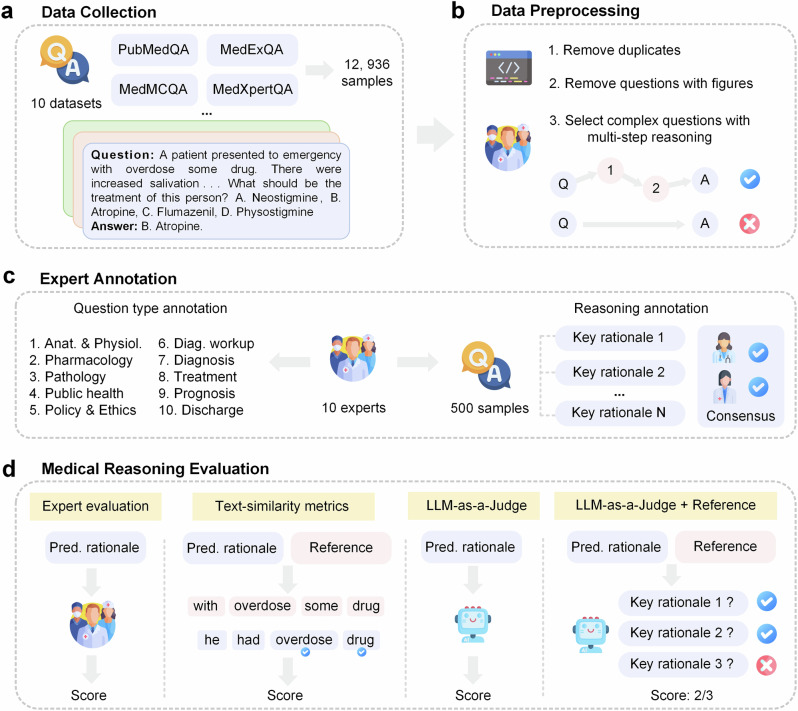
Overview of the MedThink-Bench dataset. **a** Data collection. Medical questions were sourced from ten publicly available datasets, each accompanied by ground-truth answers. **b** Data preprocessing. Duplicate entries and questions involving medical images were removed. Medical experts then manually curated a subset of complex questions requiring multi-step reasoning. **c** Expert annotation. A team of ten medical experts annotated the questions into ten distinct medical domains and collaboratively generated fine-grained reasoning trajectories through consensus. **d** Medical reasoning evaluation. We rigorously evaluated the medical reasoning capabilities of twelve LLMs, comparing them against expert evaluations, text-similarity metrics, LLM-as-a-Judge, and reference-based LLM-as-a-Judge (LLM-w-Rationale). Additionally, we analyzed the correlation between these automated metrics and expert evaluations. Icons adapted from flaticon.com, used under royalty-free license.

To address these gaps, we introduce MedThink-Bench, a benchmark tailored for rigorous, explainable, and scalable evaluation of LLMs’ medical reasoning (Fig. 1). MedThink-Bench comprises 500 challenging medical question-answer (QA) pairs across 10 representative domains, each annotated by medical professionals with fine-grained, step-by-step reasoning trajectories that mirror real-world clinical logic. Building on this resource, we propose LLM-w-Rationale, an evaluation framework that integrates the expert-curated fine-grained rationales with an LLM-as-a-Judge mechanism, thus combining their complementary strengths. Specifically, by calibrating the LLM-based evaluator with nuanced reasoning trajectories, our framework can accurately assess the intermediate reasoning to achieve expert-level factual consistency while maintaining scalability.

In this study, we demonstrate that LLM-w-Rationale correlates strongly with expert evaluation (Pearson coefficient up to 0.87) and remains robust across different prompts and judge models. Moreover, our benchmark comparison of twelve state-of-the-art LLMs reveals two surprising findings: reasoning performance shows disparity with prediction accuracy, which could truly reflect the medical capability of LLMs; smaller models such as MedGemma-27B^42^ and HuatuoGPT-o1-70B^43^ can outperform larger proprietary models like OpenAI-o3 and DeepSeek-R1^44^ in medical reasoning. Our contributions are threefold. First, we address the longstanding challenge of scalable and expert-level evaluation of LLM-generated medical rationales. Second, we construct a high-quality dataset featuring 500 expert-annotated questions with nuanced reasoning trajectories across 10 medical domains. Third, we provide a comprehensive comparison of twelve LLMs in terms of their medical reasoning capabilities. Overall, MedThink-Bench offers a foundational resource for assessing the trustworthiness of LLMs in medical decision-making, thereby advancing their safe and responsible integration into clinical practice.

## Results

We developed LLM-w-Rationale, an evaluation framework that integrates expert-curated, fine-grained rationales with the LLM-as-a-Judge paradigm to assess medical reasoning. This section summarizes the key findings, including an overview of MedThink-Bench, comparisons with conventional metrics across twelve LLMs, and analyses of the framework’s fidelity, robustness, and efficiency.

### Dataset

**Fig. 2:**
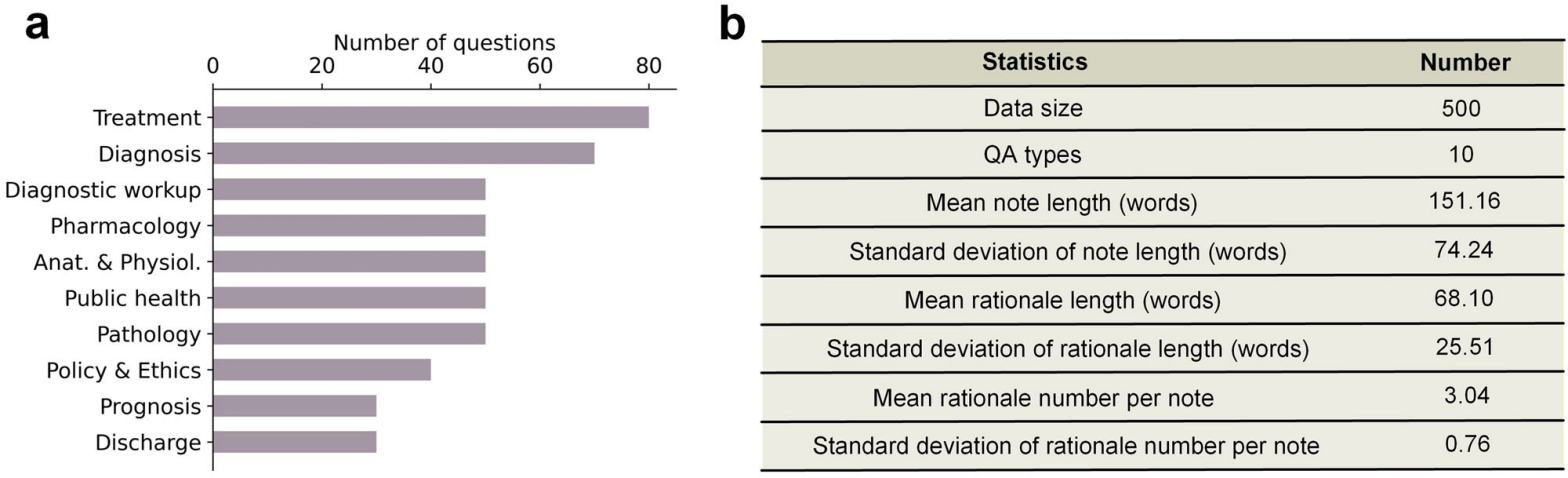
Dataset statistics of MedThink-Bench. **a** Breakdown of the 10 medical domains included in the MedThink-Bench dataset. **b** Detailed statistics of the dataset.

We created MedThink-Bench, a medical QA dataset with expert-derived reasoning annotations, comprising 500 complex questions across 10 medical domains: Pathology, Discharge, Disease Diagnosis, Anatomy & Physiology, Treatment, Public Health, Policy & Ethics, Prognosis, Diagnostic Workup, and Pharmacology. All questions were sourced from publicly available medical QA datasets (Supplementary Note 1). Our expert annotation team manually selected questions requiring multi-step reasoning and provided fine-grained annotations of the underlying reasoning processes (Supplementary Notes 2 and 3). The data statistics are presented in Fig. 2.

### Comparison of evaluation metrics on LLM reasoning

**Fig. 3:**
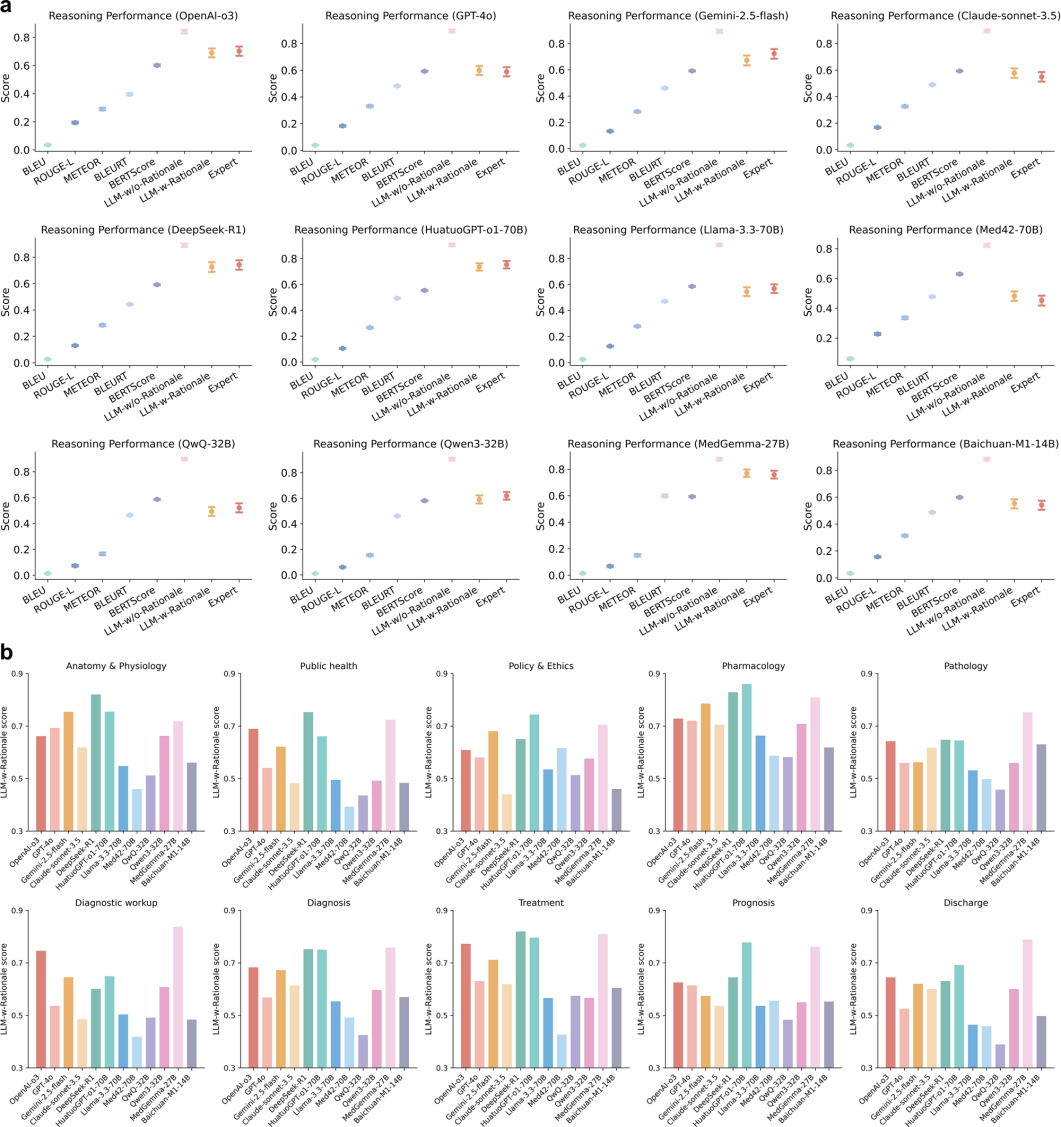
Medical reasoning performance on the MedThink-Bench dataset. **a** Comparison of overall medical reasoning performance, including expert evaluation, five text-similarity metrics, and the proposed LLM-w-Rationale framework under zero-shot prompting. The automated reasoning assessments were obtained by comparing ground-truth reasoning annotations with the predicted annotations. Error bars represent the 95% CI of the mean, calculated via bootstrapping. **b** Breakdown of medical reasoning performance across the ten medical domains in the MedThink-Bench dataset.

We evaluated the reasoning capabilities of twelve LLMs on MedThink-Bench using zero-shot Chain-of-Thought (CoT) prompting^45^ to assess their generated rationales. Performance was assessed through expert evaluations and eight automated metrics (Fig. 3a). Prediction accuracy results are provided in Supplementary Note 7. As shown in Fig. 3a, the expert evaluation scores ranged from 0.453 (95% confidence interval (CI): 0.419–0.485) for Med42-70B to 0.759 (95% CI: 0.730–0.789) for MedGemma-27B. For the reference-based LLM-as-a-Judge (LLM-w-Rationale), scores ranged from 0.482 (95% CI: 0.450–0.514) for Med42-70B to 0.769 (95% CI: 0.742–0.798) for MedGemma-27B. Meanwhile, the performance of reference-free LLM-as-a-Judge (LLM-w/o-Rationale) ranged from 0.823 (95% CI: 0.812–0.834) for Med42-70B to 0.907 (95% CI: 0.896–0.918) for Qwen3-32B. Among the text-similarity metrics, BLUERT and BERTScore generally outperformed the other metrics. Specifically, BLUERT scores ranged from 0.395 (95% CI: 0.388–0.403) for OpenAI-o3 to 0.599 (95% CI: 0.589–0.608) for MedGemma-27B, while BERTScore ranged from 0.554 (95% CI: 0.551–0.557) for HuatuoGPT-o1-70B to 0.630 (95% CI: 0.625–0.635) for Med42-70B.

### Domain-specific reasoning performance

LLM performance varied considerably across the 10 medical domains (Fig. 3b). DeepSeek-R1 achieved top performance in Anatomy & Physiology, Public Health, and Treatment, outperforming OpenAI-o3 by 0.047, 0.159, and 0.063, respectively. MedGemma-27B led in four clinically complex domains, Pathology, Diagnostic Workup, Disease Diagnosis, and Discharge, with gains over OpenAI-o3 of 0.075, 0.091, 0.110, and 0.144. HuatuoGPT-o1-70B excelled in Policy & Ethics, Pharmacology, and Prognosis, with an average margin of 0.140 over OpenAI-o3. These findings highlight that model strengths are often domain-specific, and MedThink-Bench enables fine-grained evaluation of domain-specific capabilities.

### Correlation analysis between expert and automated evaluation

**Fig. 4:**
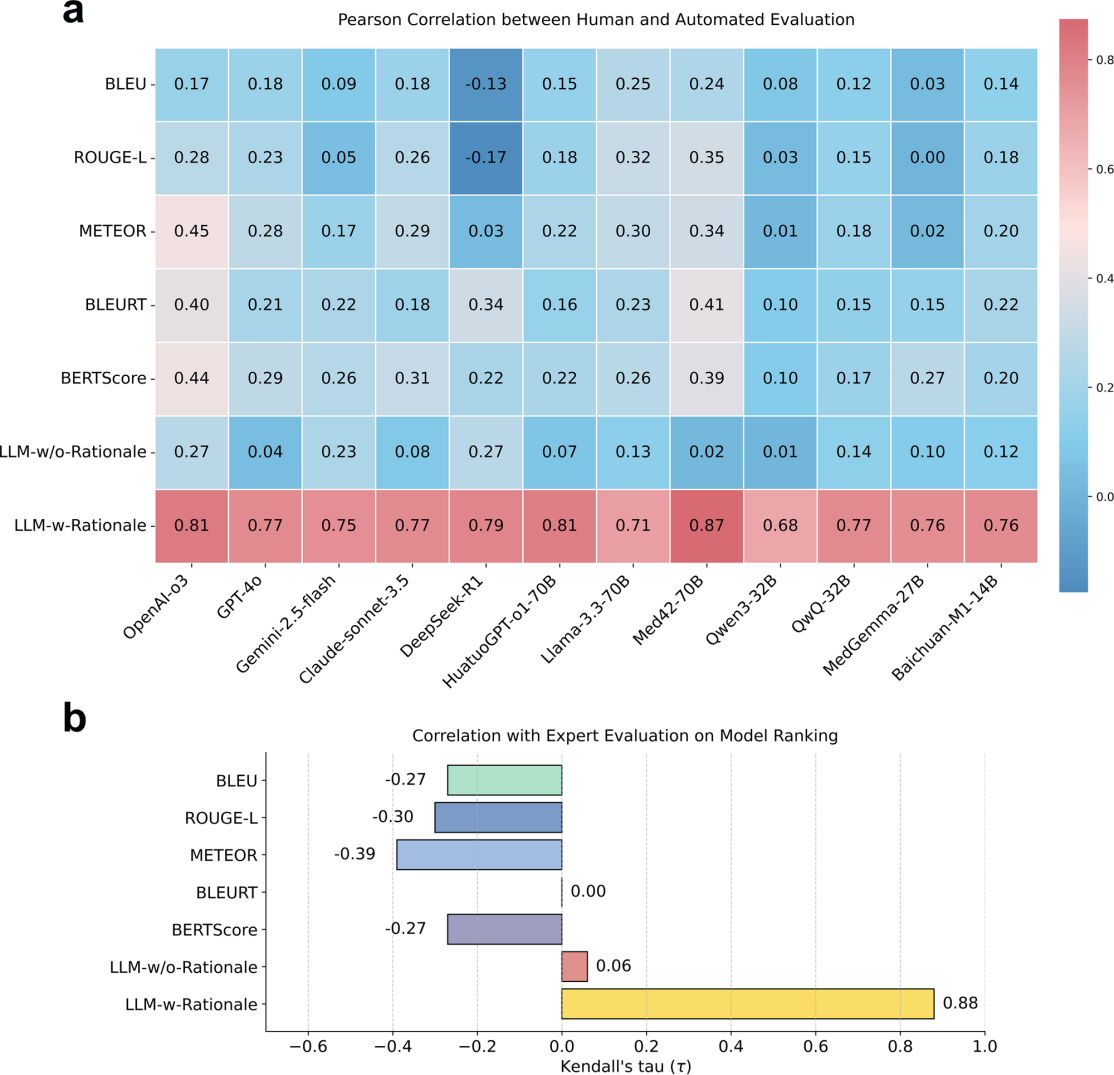
Correlation analysis between expert and automated evaluation of medical reasoning performance. **a** Pearson correlation analysis of predicted rationales against expert assessments and various automated metrics. These metrics include text-similarity measures (BLEU, ROUGE-L, METEOR, BLEURT, BERTScore), LLM-w/o-Rationale (which does not use ground-truth rationales as a reference), and LLM-w-Rationale (which uses our annotated fine-grained rationales as a reference). We take GPT-4o-mini as the judge model. Warmer colors (red tones) denote stronger positive correlations, while cooler colors (blue tones) indicate weaker or negative correlations. The results indicate a strong correlation between LLM-w-Rationale and expert evaluations, while LLM-w/o-Rationale and text-based metrics show weaker correlations with expert assessments. **b** Kendall’s tau correlation analysis on the ranking of models based on expert evaluations and the automated metrics. Using GPT-4o-mini as the judge model, LLM-w-Rationale demonstrates a very strong positive correlation (τ = 0.88) with expert rankings, whereas LLM-w/o-Rationale (τ = 0.06) and text-similarity metrics (with τ values ranging from −0.39 to 0, indicating negative or weak correlations) show much weaker associations with expert-derived model performance rankings.

**Fig. 5:**
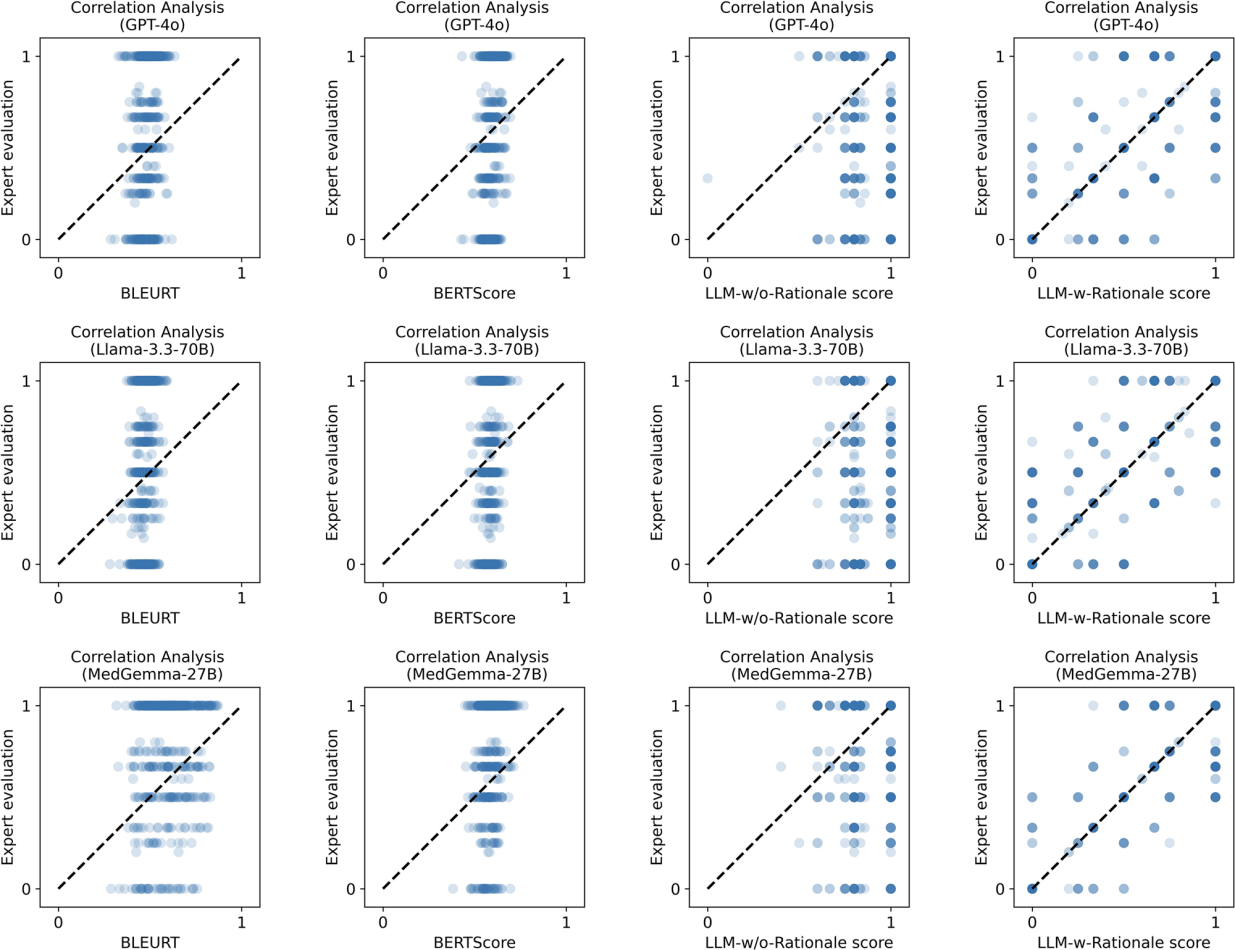
Scatter plot comparison of the scores between expert evaluation and automated metrics for each sample in the MedThink-Bench dataset. The plot includes results from GPT-4o, Llama-3.3-70B, and MedGemma-27B; the judge model in the two LLM-based evaluation metrics is GPT-4o-mini. Each point represents an individual sample, with dashed lines indicating equal performance between expert and automated scores. For LLM-w-Rationale, data points tend to align closely with the dashed line, suggesting strong agreement with expert evaluations. In contrast, BLEURT, BERTScore, and LLM-w/o-Rationale exhibit greater divergence from the dashed line, indicating a weaker alignment with expert assessments.

We computed Pearson correlation coefficients between the expert evaluation scores and all the automated metrics across all LLMs, as shown in Fig. 4a. The results reveal weak correlations for metrics such as BLEU, ROUGE-L, METEOR, BLEURT, and BERTScore, with Pearson coefficients ranging from −0.17 to 0.45. Similarly, LLM-w/o-Rationale showed weak correlation, with coefficients ranging from 0.01 to 0.27. In contrast, LLM-w-Rationale demonstrated a strong correlation with expert evaluations, with Pearson coefficients ranging from 0.68 to 0.87. We further employed Kendall’s tau correlation to assess the concordance between model rankings derived from expert evaluations and those obtained from automated metrics (Fig. 4b). Both LLM-w/o-Rationale (τ = 0.06) and text-similarity metrics (τ ranging from −0.39 to 0) exhibited weak or negative associations with expert-derived rankings. In contrast, LLM-w-Rationale achieved a markedly strong positive correlation with expert assessments (τ = 0.88), indicating its close alignment with human judgment. Additionally, we visualized the individual evaluation scores of the LLMs in Fig. 5 and Supplementary Note 8. The results demonstrated that data points for LLM-w-Rationale generally closely align with the dashed line, indicating agreement with expert scores. In contrast, BLEURT, BERTScore, and LLM-w/o-Rationale show greater divergence from expert evaluations.

### Stratified discrimination analysis of evaluation metrics

**Fig. 6:**
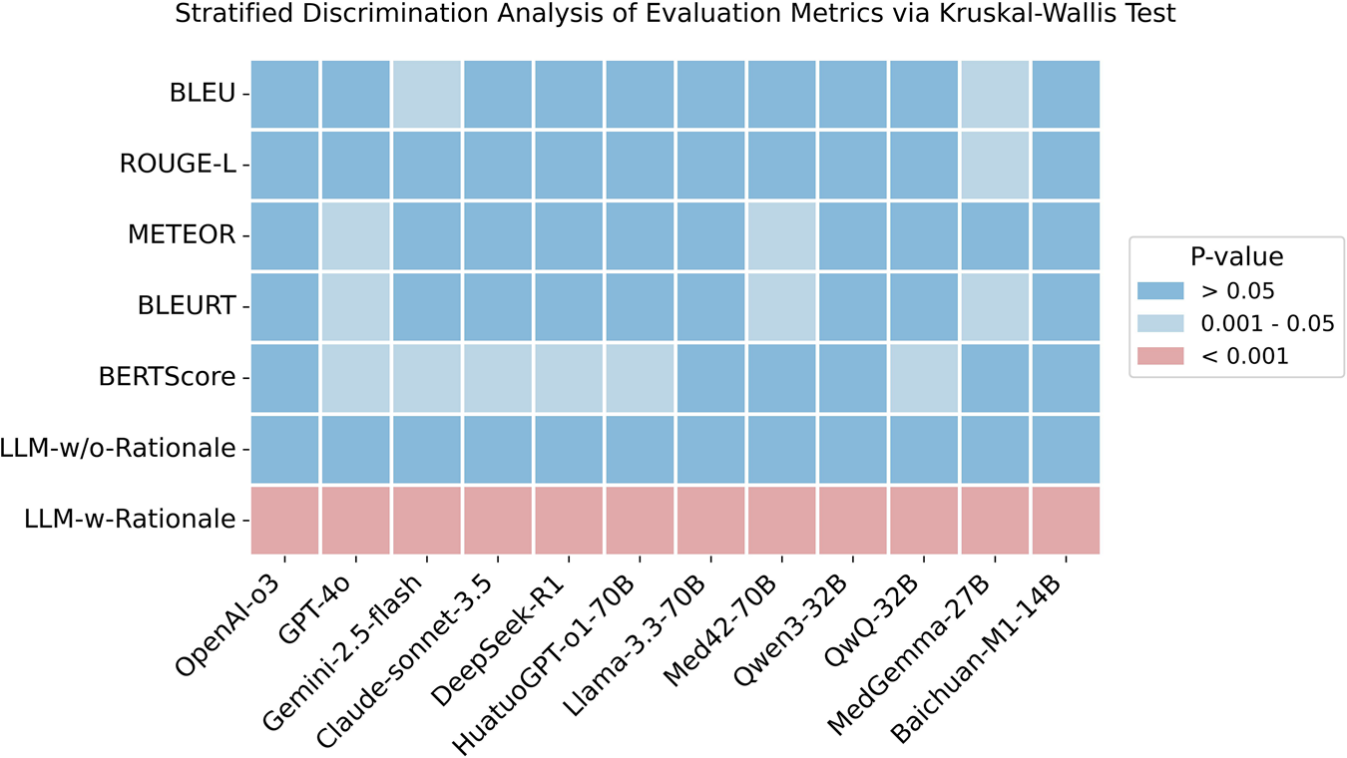
Heatmap of p-values obtained from Kruskal–Wallis H tests evaluating the stratified discrimination power of semantic similarity metrics. Human-evaluated samples are grouped into three quality levels: “low score,” “medium score,” and “high score.” For each metric, the test evaluates whether the score distributions significantly differ across these quality strata. Lower p-values (e.g., *p* < 0.001) indicate stronger discriminative power, reflecting the metric’s ability to distinguish between different levels of medical reasoning quality.

Discriminative power is a key property of an effective evaluation metric. To assess this, we conducted a stratified discrimination analysis, categorizing data samples into three quality levels, i.e., low, medium, and high, based on human evaluation scores. Figure 6 presents a heatmap of p-values from Kruskal–Wallis H tests applied to each evaluation metric. Traditional text similarity metrics such as BLEU and ROUGE-L, as well as LLM-w/o-Rationale, demonstrated limited discriminative capability, with several p-values exceeding the 0.05 threshold, indicating low sensitivity to quality differences. In contrast, LLM-w-Rationale, which incorporates fine-grained rationales, consistently yielded the lowest p-values (*p* < 0.001) across multiple judge models, highlighting its superior ability to distinguish among the three quality levels.

### Impact of the judge model in LLM-w-Rationale

**Fig. 7:**
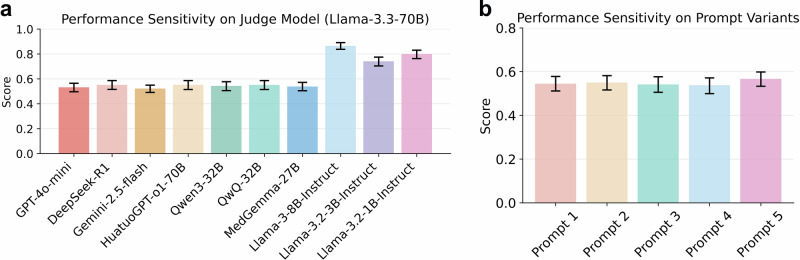
Performance robustness analysis of LLM-w-Rationale. **a** Robustness of LLM-w-Rationale across different judge models. Predicted rationales from Llama-3.3-70B were evaluated using 10 LLMs of varying scales as judge models. **b** Sensitivity of LLM-w-Rationale to prompt variations. Five semantically similar prompt variations were tested to assess the framework’s robustness. Error bars represent the 95% CI of the mean, calculated via bootstrapping.

We evaluated the robustness of LLM-w-Rationale to the judge model. Specifically, predicted rationales generated by four LLMs (Llama-3.3-70B, OpenAI-o3, Qwen3-32B, and MedGemma-27B) were assessed using 10 different LLMs serving as judge models (Fig. 7a and Supplementary Note 10). As shown in Fig. 7a, when rationales predicted by Llama-3.3-70B were evaluated, the reasoning scores ranged from 0.522 (95% CI: 0.492–0.550) for Gemini-2.5-flash to 0.553 (95% CI: 0.515–0.587) for HuatuoGPT-o1-70B. However, when using smaller models as the judge models, such as Llama-3-8B-Instruct, Llama-3.2-3B-Instruct, and Llama-3.2-1B-Instruct, the performance is higher than that of the large models, which range from 0.704 to 0.891.

### Prompt sensitivity in LLM-w-Rationale

We also examined the sensitivity of the LLM-w-Rationale framework to different prompt formulations by composing five prompt variations with similar semantics (Supplementary Note 9). The reasoning performance of each variation was compared using the predicted rationales from Llama-3.3-70B, with GPT-4o-mini fixed as the judge model. As shown in Fig. 7b, the performance varied from 0.538 (95% CI: 0.500–0.571) for the second prompt variant to 0.567 (95% CI: 0.533–0.599) for the fifth prompt.

### Efficiency comparison

To evaluate scalability, we compared the time efficiency of different evaluation strategies. Specifically, we recorded the running times for human evaluation, text-similarity metrics, and LLM-w-Rationale evaluations on MedThink-Bench. As shown in Table 1, the average assessment time for text-similarity metrics was 9.0 min, while LLM-w-Rationale took 310.7 min when using HuatuoGPT-o1-70B as the judge model. In contrast, human evaluation required an average of 3708.3 min, significantly longer than the automated metrics.

**Table 1:** Comparison of running time and cost for rationale assessment among text-similarity metrics, LLM-w-Rationale, and expert evaluation on the MedThink-Bench dataset

Evaluation	Time (minutes)	Cost ($)
Text-similarity metric	9.0 ± 0.6	0
LLM-w-Rationale (GPT-4o-mini)	51.8 ± 5.6	0.8 ± 0.1
LLM-w-Rationale (HuatuoGPT-o1-70B)	310.7 ± 14.1	0
LLM-w-Rationale (MedGemma-27B)	74.8 ± 8.9	0
Expert evaluation	3708.3 ± 175.3	NA

Running time for text-similarity metrics represents the cumulative time across all evaluated metrics. Cost calculations are limited to API service fees; GPU running costs are excluded, as local GPUs on a Linux server were utilized for experiments without incurring additional hardware operation expenses.

*NA* not applicable.

### Case study

**Fig. 8:**
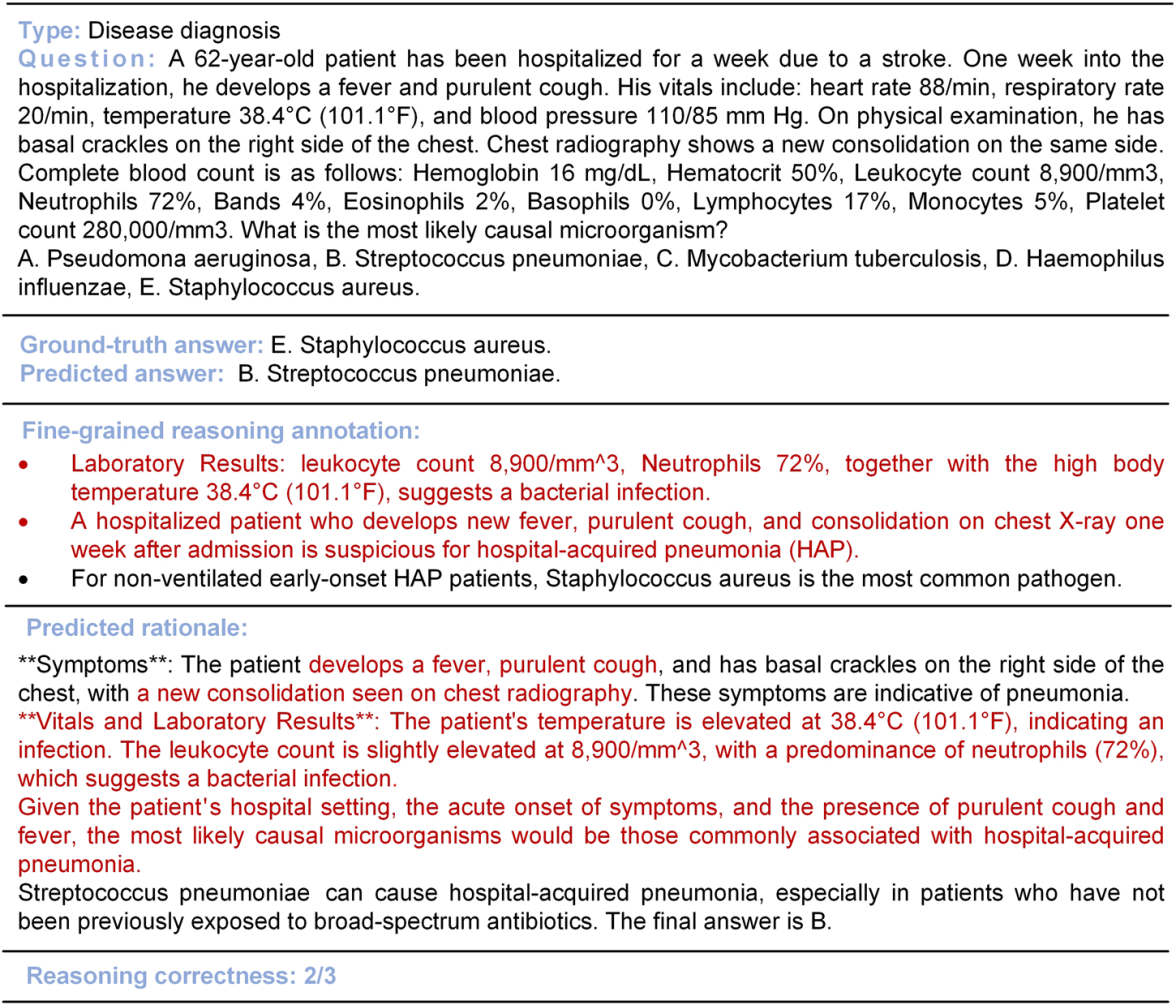
Case study of medical reasoning assessment. This case demonstrates that while the prediction model Llama-3.3-70B produced an incorrect answer, it followed partially correct medical reasoning trajectories (highlighted in red). This underscores the advantage of the LLM-w-Rationale framework, which, in conjunction with the fine-grained rationale annotations in MedThink-Bench, provides a more nuanced evaluation of the medical reasoning abilities of LLMs compared to merely assessing prediction accuracy.

Case studies were conducted to demonstrate the effectiveness of LLM-w-Rationale in measuring the medical reasoning capability of LLMs. As shown in Fig. 8, Llama-3.3-70B produced an incorrect answer, yet followed a partially correct medical reasoning trajectory. In another case (Supplementary Note 11), flawed reasoning led to a correct answer. By measuring medical reasoning with expert-curated fine-grained rationales, LLM-w-Rationale captured flawed reasoning patterns and offered a more nuanced evaluation of an LLM’s medical capabilities. These findings underscore the importance of assessing not only the final prediction but also the underlying reasoning process.

### Correlation between reasoning performance and prediction accuracy

**Fig. 9:**
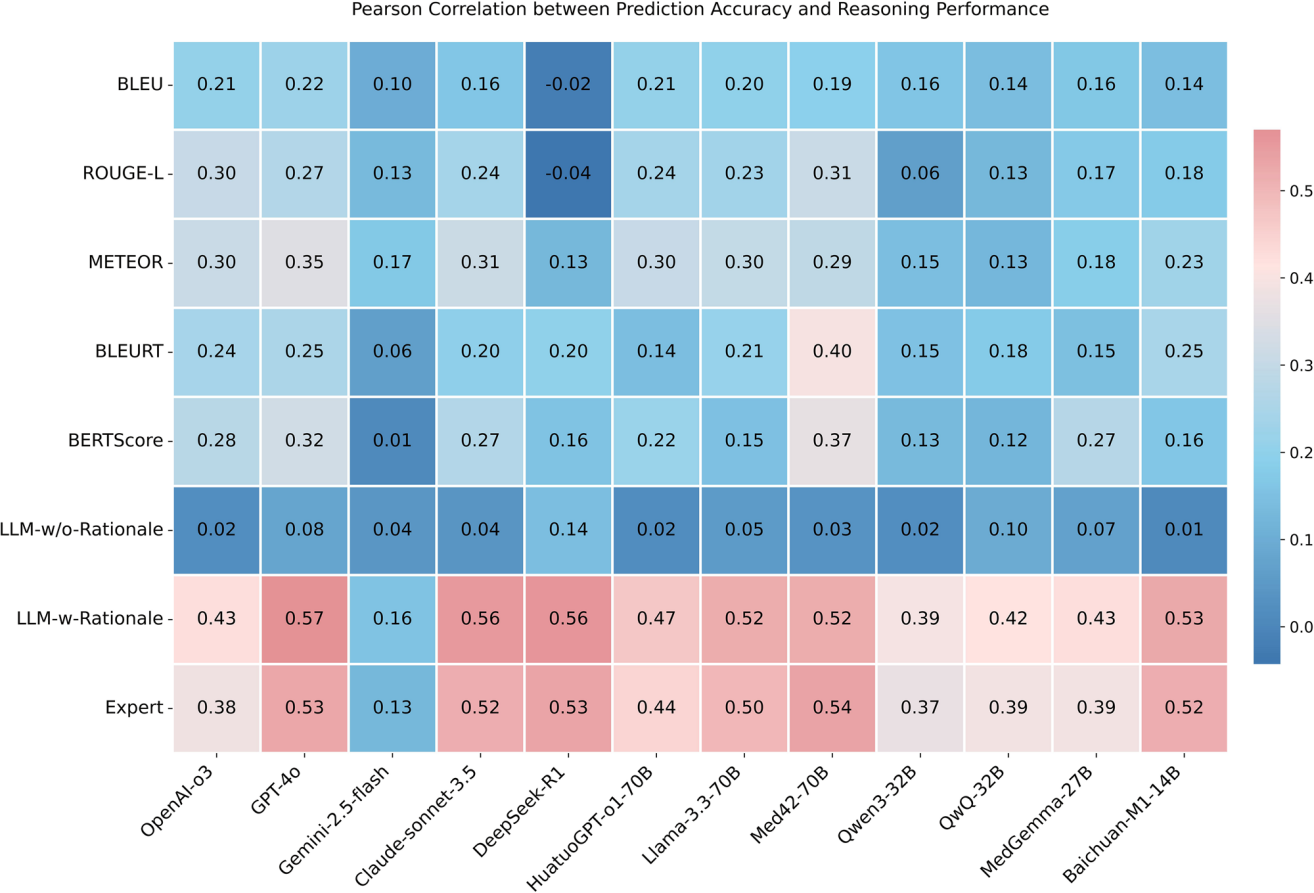
Pearson correlation between prediction accuracy and the reasoning performance across various evaluation metrics. Warmer colors (red tones) denote stronger positive correlations, while cooler colors (blue tones) indicate weaker or negative correlations. Most automated metrics (e.g., BLEU, ROUGE-L, METEOR) show weak to moderate positive correlations with prediction accuracy. Overall, both LLM-w-Rationale and expert evaluation exhibit higher positive correlations with prediction accuracy, though not strong. This finding aligns with observations that prediction accuracy alone inadequately captures reasoning quality, as correct multiple-choice answers may stem from flawed reasoning and incorrect answers can contain partially valid rationale.

We computed Pearson correlation coefficients between the medical QA prediction accuracy and reasoning evaluation metrics (Fig. 9). The results revealed weak or moderate correlations for text-similarity metrics and LLM-w/o-Rationale, with coefficients ranging from −0.04 to 0.40. Similarly, both LLM-w-Rationale and expert evaluation exhibited moderate correlations with expert assessments, with average Pearson correlation coefficients of 0.462 and 0.436, respectively.

### Analysis of data leakage

**Fig. 10:**
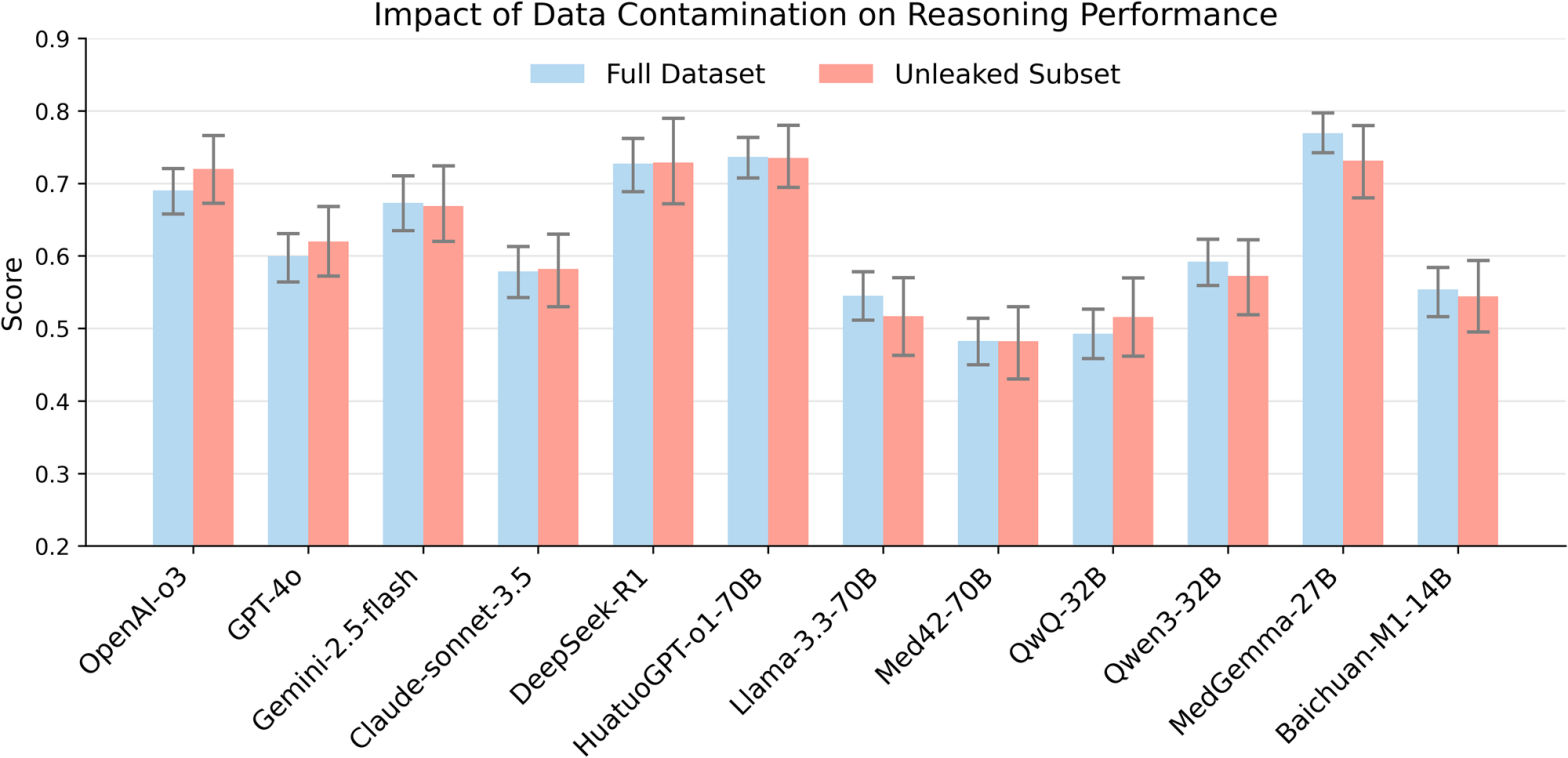
Impact of data contamination on reasoning evaluation. The bar plot illustrates reasoning scores obtained via LLM-w-Rationale on the full dataset (blue bars) and an uncontaminated subset (red bars). Error bars represent the 95% CI of the mean, calculated via bootstrapping. Reasoning performance shows minimal variation between the full dataset and the uncontaminated subset across models, indicating limited influence of data leakage on reasoning evaluation.

Given that MedThink-Bench was constructed from publicly available datasets, we examined potential data contamination that might have occurred during LLM pre-training and could influence evaluation outcomes. Since the rationale annotations were independently curated by medical experts, their leakage was not assessed. As shown in Table 2, MedGemma-27B and Llama-3.3-70B exhibited relatively high data contamination ratios of 0.252 and 0.118, respectively, substantially higher than those observed for Qwen3-32B, Baichuan-M1-14B, and HuatuoGPT-o1-70B. These results indicated that the training data of certain LLMs, particularly MedGemma-27B and Llama-3.3-70B, likely included portions of public medical QA datasets. To evaluate the impact of contamination on reasoning performance and QA performance, we further analyzed a subset of 200 uncontaminated samples. As shown in Fig. 10 and Supplementary Fig. 1, reasoning performance remained largely consistent across all models, indicating minimal influence from data leakage. In contrast, QA prediction accuracy declined notably in a few models (e.g., MedGemma-27B and Llama-3.3-70B). Overall, these findings suggest that while data contamination may affect QA prediction to some extent, it does not substantially influence the reasoning evaluation or alter the main conclusions of this study.

**Table 2:** Estimated data contamination rates of MedThink-Bench in investigated LLMs

Prediction model	Data contamination rate
GPT-4o	0.004
Gemini-2.5-flash	0.032
Claude-sonnet-3.5	0.054
DeepSeek-R1	0.048
HuatuoGPT-o1-70B	0.000
Llama-3.3-70B	0.118
Med42-70B	0.016
Qwen3-32B	0.038
QwQ-32B	0.034
MedGemma-27B	0.252
Baichuan-M1-14B	0.076

Lower values indicate a lower likelihood of data contamination. Contamination for OpenAI-o3 was not assessed because the model does not permit temperature control, which is required by the contamination detection method.

### Error analysis of reasoning evaluation

**Fig. 11:**
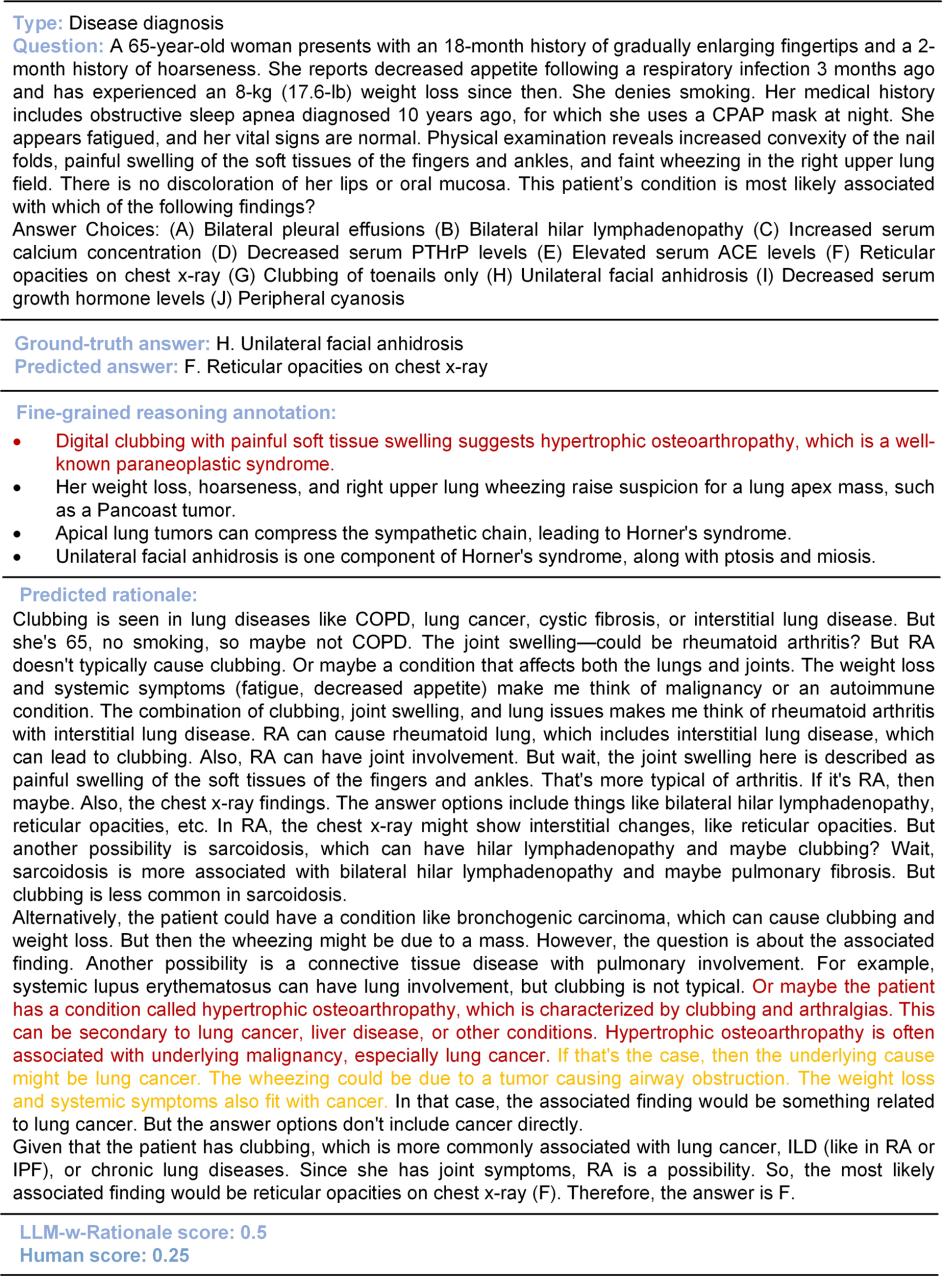
Error analysis of a false-positive case produced by LLM-w-Rationale on medical reasoning evaluation. The prediction was generated by Qwen3-32B and evaluated by the GPT-4o-mini judge model. Correct reasoning trajectories are highlighted in red, and the false-positive trajectory is highlighted in yellow.

We conducted an error detection analysis to quantify the degree of divergence between the LLM-w-Rationale framework and expert evaluations. Expert assessments of rationale correctness were used as the ground truth. As summarized in Table 3, the proposed LLM-w-Rationale framework demonstrated strong concordance with expert evaluation. Across 12 tested models, Precision, Recall, and F_1_-scores were all ≥0.755, with overall averages of 0.849, 0.839, and 0.843, respectively. These results indicate that the framework makes only minor classification errors and maintains high consistency with human judgment in medical reasoning assessment. To further understand sources of disagreement, we analyzed representative error cases where LLM-w-Rationale’s evaluations diverged from expert assessments. As shown in Fig. 11, a false-positive occurred when LLM-w-Rationale assigned a higher score than experts because it treated a tentative reasoning path as correct, overlooking its later rejection. Conversely, a false-negative case (Supplementary Note 12) arose when the judge model lowered the score, assuming key information was missing, while experts agreed that the reasoning contained essential elements and should be marked correct.

**Table 3:** Error detection performance of the LLM-w-Rationale framework against expert evaluation (ground truth) for medical reasoning assessment

Model	Precision	Recall	F_1_
OpenAI-o3	0.902	0.884	0.893
GPT-4o	0.826	0.839	0.832
Gemini-2.5-flash	0.884	0.822	0.852
Claude-sonnet-3.5	0.812	0.850	0.831
DeepSeek-R1	0.899	0.878	0.889
HuatuoGPT-o1-70B	0.910	0.887	0.899
Llama-3.3-70B	0.800	0.764	0.781
Med42-70B	0.815	0.881	0.847
QwQ-32B	0.820	0.769	0.794
Qwen3-32B	0.805	0.755	0.779
MedGemma-27B	0.900	0.922	0.911
Baichuan-M1-14B	0.809	0.815	0.812
Average	0.849	0.839	0.843

## Discussion

In this study, we presented MedThink-Bench, a curated dataset comprising complex medical questions spanning 10 clinical scenarios, each accompanied by fine-grained rationale annotations from domain experts. MedThink-Bench was designed to address a critical challenge in evaluating LLMs for medical reasoning: enabling automated assessment while maintaining expert-level factual accuracy. The key findings and insights are summarized below.

First, MedThink-Bench effectively differentiated the reasoning capabilities of the LLMs (Fig. 3a). Our benchmarking results showed that MedGemma-27B, HuatuoGPT-o1-70B, and DeepSeek-R1 achieved the highest overall reasoning performance. Surprisingly, smaller open-source models like MedGemma-27B outperformed larger commercial counterparts, including OpenAI-o3 and DeepSeek-R1. Likewise, Qwen3-32B significantly outperformed Gemini-2.5-flash and DeepSeek-R1 (*p* < 0.001). Among commercial LLMs, DeepSeek-R1, OpenAI-o3, and Gemini-2.5-flash emerged as the top performers, substantially surpassing GPT-4o and Claude-sonnet-3.5. The domain-wise breakdown (Fig. 3b) further highlighted nuanced differences in model capabilities across clinical areas, offering practical guidance for model selection in clinical applications.

Second, the proposed evaluation strategy, LLM-w-Rationale, exhibited strong alignment with expert evaluations (Fig. 4a, b). Specifically, the Pearson correlation coefficients between LLM-w-Rationale and expert scores ranged from 0.68 to 0.87, while Kendall’s tau correlation for model ranking reached τ = 0.88, both indicating a strong concordance with expert judgment. Visualization of the score distributions (Fig. 5) further confirmed this consistency. Importantly, LLM-w-Rationale demonstrated significantly higher efficiency compared to human evaluation (Table 1) and was cost-effective, making it well-suited for scalable, automated evaluation. Together, these results demonstrated that integrating fine-grained rationale annotations as references within the LLM-as-a-Judge paradigm enabled efficient, reliable, and expert-aligned evaluation of medical reasoning.

Third, our analysis revealed that conventional reasoning evaluation methods, including text similarity metrics and LLM-w/o-Rationale, showed weak correlation with expert judgments (Fig. 4a). This was primarily because metrics such as BLEU and ROUGE relied on surface-level word overlap and failed to capture semantic or logical equivalence. Although BERTScore incorporated word embeddings, it remained limited in two key aspects. First, it operates at the word level rather than reasoning-chain level. Second, it cannot comprehend the underlying logical structure of complex medical justifications. As a result, these metrics often produced narrow score ranges (Fig. 5), since LLMs tended to repeat contextual information, generating superficially plausible rationales that were misinterpreted as accurate (Supplementary Figs. 5 and 6). In contrast, LLM-w/o-Rationale lacked access to ground-truth rationales and highly depended on the judge model’s capacity and biases, undermining its reliability. Overall, these findings underscore the limitations of existing strategies for assessing medical reasoning performance.

Additionally, LLM-w-Rationale exhibited superior discriminative ability in evaluating reasoning quality (Fig. 6). It assigned significantly different scores to outputs of the three human-rated quality levels (low, medium, and high scores), demonstrating its ability to distinguish reasoning levels. In contrast, text-similarity metrics, such as METEOR and BERTScore, and LLM-w/o-Rationale failed to make such distinctions. These findings empirically support the discriminative validity of the LLM-w-Rationale as a more reliable, human-aligned evaluation method for medical reasoning.

We further demonstrated that LLM-w-Rationale was robust to variations in both the judge model and prompt phrasing. As shown in Fig. 7a and Supplementary Fig. 3, evaluation outcomes remained stable when using LLMs with strong instruction-following capabilities, such as GPT-4o-mini or MedGemma-27B, as judge models. This robustness likely stems from these models’ ability to faithfully execute instructions (Supplementary Note 6), such as rejecting rationales that deviate from or contradict the reference standard (Supplementary Figs. 5 and 6). To secure evaluation reliability, we recommend selecting judge models with demonstrated instruction-following proficiency. Likewise, results in Fig. 7b indicated that prompt variants with similar semantics yielded consistent evaluation outcomes, suggesting robustness to prompt engineering. This stability enhanced the practical applicability of LLM-w-Rationale for widespread use in reasoning evaluation.

Finally, we observed a discrepancy between reasoning performance and prediction accuracy (Figs. 3a, b and 9, and Supplementary Fig. 1). For instance, while OpenAI-o3 underperformed MedGemma-27B and HuatuoGPT-o1-70B in reasoning (Fig. 3a, b), it achieved the highest prediction accuracy at 0.692 (95% CI: 0.652–0.732), compared to 0.384 (95% CI: 0.342–0.426) and 0.49 (95% CI: 0.446–0.532), respectively. This divergence could be attributed to two factors. First, incorrect multiple-choice answers sometimes contained partially valid reasoning, which was captured by rationale-based evaluation but not prediction accuracy (Fig. 8). Second, some LLMs produced correct multiple-choice answers through flawed reasoning, leading to inflated accuracy despite weak justifications (Supplementary Figs. 5 and 6). These findings underscore the limitations of the prediction accuracy in evaluating medical reasoning. In contrast, rationale-based evaluation provides a more nuanced and faithful representation of an LLM’s reasoning quality, offering deeper insights into its alignment with expert-level thinking.

Despite these advances, our study has several limitations. First, the LLM-w-Rationale framework currently assesses only two critical dimensions of reasoning: correctness and comprehensiveness, while other dimensions, such as fairness, potential harm, and readability, remain unexplored^14,46^. Future research should investigate methods to systematically evaluate these additional dimensions to better align with expert-level expectations. Second, although LLM-w-Rationale correlates highly with expert evaluation (Figs. 4a, b and 5), discrepancies remain, manifesting as false-negative or false-positive cases (Fig. 11 and Supplementary Fig. 7). These could potentially be mitigated by employing a more capable judge model with stronger instruction-following abilities^47^ or by integrating advanced prompt-engineering strategies^23,26^. Third, our evaluation framework relies on expert-curated annotations as reference standards, which are labor-intensive and consequently limit the size of the MedThink-Bench dataset. Future work may explore reference-free approaches to achieve high-fidelity and scalable assessments.

In summary, this study addressed the critical challenge of accurately and efficiently evaluating the medical reasoning capabilities of LLMs. To this end, we curated MedThink-Bench, a medical QA dataset covering 10 clinical scenarios with fine-grained rationale annotations from domain experts. We introduced an evaluation framework, LLM-w-Rationale, that combines the nuanced annotations with the LLM-as-a-Judge paradigm to enable automated yet expert-aligned reasoning assessment. Using this framework, we benchmarked the reasoning performance of twelve LLMs and systematically compared multiple evaluation strategies. Our findings demonstrated that LLM-w-Rationale achieved strong concordance with expert assessments while offering substantially greater efficiency. Overall, this work provided a robust and scalable solution for evaluating LLMs’ medical reasoning, reducing the burden of manual evaluation and paving the way for their integration into clinical practice.

## Methods

### Data curation

We collected comprehensive medical questions from 10 existing datasets, filtered out hard questions that required multi-step reasoning (Supplementary Note 2), and annotated the reasoning rationales by experts. Specifically, the medical QA datasets from which we collected questions included MedBullets^48^, MMLU-Pro^49^, MedExQA^50^, MedXpertQA^21^, Humanity’s Last Exam^51^, MedQA-USMLE^52^, PubMedQA^53^, MedMCQA^54^, MMLU-Medicine^55^, and HEAD-QA^56^. The statistics of these datasets are shown in Fig. 2. Then, we pre-processed the collected data by removing duplicates and filtering out the questions involving images for prediction.

### Data annotation

We built a well-annotated dataset to facilitate automated expert-level reasoning evaluation. The medical questions were divided into 10 medical domains: Pathology, Discharge, Disease Diagnosis, Anatomy & Physiology, Treatment, Public Health, Policy & Ethics, Prognosis, Diagnostic Workup, and Pharmacology. We employed 10 medical experts to curate the dataset manually. Two independent physicians annotated each medical question. When disagreement existed in the annotation, a third physician examined the case and made the final annotation. We checked the inter-annotator agreement on the reasoning and question types (Supplementary Note 3).

### Evaluation framework

To evaluate medical rationales, we applied three evaluation strategies: human evaluation, LLM-as-a-Judge, and text-similarity metrics. Notably, we proposed an evaluation framework, LLM-w-Rationale, that enabled scalable, step-level assessment of clinical reasoning abilities of LLMs. Built upon MedThink-Bench’s nuanced rationales, LLM-w-Rationale compared model-generated rationales against expert-annotated reasoning trajectories to evaluate their logical correctness and stepwise completeness. Additionally, the prediction accuracy was computed to compare LLMs’ medical capability. The following section describes the implementation and evaluation protocol in detail.

To validate the reliability of our evaluation framework, we conducted a human evaluation and compared its results with those of automated metrics. Specifically, for each question, domain experts were provided with the medical question, answer, model-generated rationale, and a grading scheme (Supplementary Note 5), which functioned as a protocol to ensure consistency and uniformity in assessment. Experts examined each rationale step-by-step, recording (1) the number of reasoning steps they considered necessary to reach the correct answer for that question, and (2) how many of those necessary steps were present in the model’s rationale. The instance-level reasoning score was defined as:

1 $${R}^{\left(i\right)}=\frac{{ExpertCovered}{(r}_{{model}}^{\left(i\right)},{q}^{\left(i\right)})}{{ExpertRequired}\left({q}^{\left(i\right)}\right)}$$

Here ${r}_{{model}}^{(i)}$ is the model-generated rationale for the question ${q}^{\left(i\right)}$, and ${ExpertRequired}({q}^{(i)})$ is the number of reasoning steps the experts deem necessary for that question in real-time assessment, while ${ExpertCovered}{(r}_{{model}}^{(i)},{q}^{(i)})$ denotes the number of those required steps that experts judge to appear in the model’s rationale.

To achieve fine-grained and scalable evaluation aligned with expert reasoning, we adopted a reference-based LLM-as-a-Judge approach (LLM-w-Rationale) that operates at the reasoning-step level. For each question, expert-annotated rationales consist of discrete reasoning steps provided by medical specialists during dataset construction. To assess step-level correctness, the judge model receives the question, the model-generated rationale (as a whole), and each expert reasoning step individually. It is then prompted to determine whether the generated rationale adequately supports each expert step. This design enables robust evaluation even when the model and expert rationales differ in length or granularity. Instead of enforcing step-by-step alignment, we adopt a one-to-many comparison, where each expert step is independently matched against the complete model rationale. This grading scheme differs from prior studies and is customized for complex medical reasoning evaluation. The instance-level reasoning score is calculated as the proportion of expert steps that are successfully supported by the model rationale:

2 $${R}^{\left(i\right)}=\frac{|\left\{s\in {S}_{{expert}}^{\left(i\right)}\right|{LLMJudge}(s,{r}_{{model}}^{\left(i\right)},{q}^{\left(i\right)})={Yes}\}|}{|{{S}^{\left(i\right)}}_{{expert}}|}$$

Here ${{S}^{(i)}}_{{expert}}$ is the set of expert-annotated reasoning steps for the question ${q}^{\left(i\right)}$, and ${{r}^{(i)}}_{{model}}$ is the model-generated rationale, as previously defined.

To examine the necessity of expert-annotated rationales for accurate evaluation, we implemented a commonly adopted baseline: LLM-as-a-Judge without reference. This setting allowed us to compare grounded and ungrounded evaluations and to better understand the role of reference rationales in ensuring the reliable assessment of clinical reasoning. Specifically, in this setting, the judge model was provided only with the medical question, answer, and the model-generated rationale. The judge model was first asked to estimate the number of reasoning steps required to answer the question, and then to determine how many of those steps were sufficiently supported by the model-generated rationale (Supplementary Note 6). The instance-level score was calculated as:

3 $${R}^{\left(i\right)}=\frac{{LLMCovered}{(r}_{{model}}^{\left(i\right)},{q}^{\left(i\right)})}{{LLMRequired}\left({q}^{\left(i\right)}\right)}$$

${LLMRequired}\left({q}^{\left(i\right)}\right)$ denotes the number of reasoning steps the LLM estimates are necessary to answer the question ${q}^{\left(i\right)}$, ${LLMCovered}\left({r}_{{model}}^{(i)},{q}^{(i)}\right)$ and indicates how many of those steps the LLM judges to be sufficiently reflected in the rationale.

To contextualize our evaluation framework, we reported baseline performance using widely adopted metrics, including BLEU^32^, ROUGE-L^33^, METEOR^57^, BLEURT^58^, and BERTScore^34^. Metrics such as BLEU and ROUGE-L rely on surface-level token overlap, using n-gram precision or longest common subsequence to quantify similarity. While computationally efficient, they are insensitive to paraphrasing and semantic equivalence. More recent metrics like METEOR, BLEURT, and BERTScore incorporate semantic information through synonym matching, pretrained language models, or human-annotated supervision. Although these approaches better capture general linguistic similarity, they remain limited in evaluating factual accuracy, logical soundness, and clinical relevance. While these metrics offer convenient and scalable evaluation, they do not explicitly assess the logical structure, clinical validity, or step-by-step correctness of medical reasoning. Therefore, they are used here primarily for baseline comparison.

For all step-level evaluation methods described above, we reported both instance-level and dataset-level scores. Given instance-level reasoning scores $R(i)$ for each question, $i=1,...,N,$ the final dataset-level score was computed as the average:

4 $$R=\frac{1}{N}{\sum }_{i=1}^{N}{R}^{\left(i\right)}$$

Notably, an effective reasoning evaluation should faithfully reflect the model’s genuine performance rather than pursue higher absolute “scores.” Therefore, we prioritized the evaluation methods that best aligned with human assessments, rather than those yielding the highest numerical results.

Apart from reasoning evaluation, we followed related studies and evaluated the performance of the predicted answer with accuracy using an exact match. Formally, the instance-level accuracy is:

5 $${A}^{\left(i\right)}=1\left({{a}_{{pred}}}^{\left(i\right)}={{a}_{{gold}}}^{\left(i\right)}\right)$$

where ${{a}_{{pred}}}^{(i)}$ and ${{a}_{{gold}}}^{(i)}$ denotes the predicted and golden answer for the *i*th question, the indicator function 1(⋅) returns 1 if the condition is satisfied, and 0 otherwise. The final accuracy is the average over the dataset:

6 $$A=\frac{1}{N}{\sum }_{i=1}^{N}{A}^{\left(i\right)}$$

For each sample in MedThink-Bench, we extracted the model-generated rationale and final answer using rule-based regular expressions. In the human evaluation, 10 medical professionals assessed whether each annotated reasoning step was present in the generated rationale. For automatic evaluation, we employed GPT-4o-mini as the judge model (LLM-w-Rationale), with prompts detailed in Supplementary Note 6. The model received the medical question, the generated rationale, and the fine-grained reasoning trajectories. Decoding was performed with a temperature of 0.1, a commonly adopted setting in LLM-as-a-Judge evaluations^59^, using a maximum decoding length of 4096 tokens and a fixed random seed (42) for reproducibility. In the reference-free setting, all parameters remained unchanged. For text similarity evaluation, we computed BLEU, ROUGE-L, METEOR, BERTScore, and BLEURT using standard toolkits with default configurations.

### LLM baselines

We benchmarked representative LLMs from both commercial closed-source LLMs and open-source LLMs. Since this work aimed to assess the medical reasoning capabilities of LLMs, we included both reasoning and non-reasoning LLMs from each category. Specifically, the commercial closed-source LLMs included GPT-4o^60^, OpenAI-o3, Claude-3.5-sonnet^61^, Gemini-2.5-flash^62^, DeepSeek-R1^44^, and open-source LLMs include Baichuan-M1-14B^63^, HuatuoGPT-o1-70B^64^, MedGemma-27B^42^, Med42-70B^65^, Llama-3.3-70B^66^, Qwen3-32B^67^, and QwQ-32B^68^. In the experiment, we obtained responses from closed-source LLMs through APIs. For open-source LLMs, we used the package vLLM to load them for faster inference. For inference, we adopted zero-shot CoT to prompt LLMs to generate medical reasoning (Supplementary Note 4).

The LLM inference configuration employs carefully selected hyperparameters to ensure optimal performance and reproducibility. The temperature parameter is set to 0, implementing deterministic sampling to eliminate randomness in token selection and ensure consistent outputs across multiple runs. A fixed random seed of 42 is specified to guarantee reproducible results, which is essential for scientific rigor and experimental validation. The maximum token limit is configured to 4096, providing sufficient generation capacity for comprehensive reasoning. These parameter settings collectively establish a controlled inference environment that prioritizes consistency and reliability over creative variation, aligning with the systematic evaluation requirements of our benchmarking framework.

### Stratified discrimination analysis

To assess the discriminative power of each evaluation metric, we conducted a stratified discrimination analysis, which evaluates how well a metric distinguishes between system outputs of varying quality. Specifically, the data samples were stratified into three quality levels, low (0.1–0.4), medium (0.4–0.6), and high (0.6–0.9), based on the human evaluation scores. Then, for each evaluation metric, we computed the average metric scores within each human quality stratum. To statistically assess whether the metric scores significantly differ across the three quality levels, we applied the Kruskal–Wallis H test^69^. Lower p-values (especially those <0.001) indicate stronger stratified discrimination capability, that is, the metric assigns significantly different scores to outputs of different human-rated quality levels. Metrics with better stratified discrimination can more reliably reflect human judgment and are therefore more suitable for practical evaluation scenarios.

### Data leakage analysis

To ensure MedThink-Bench evaluated clinical reasoning rather than memorized content, we adopted the CDD framework^70^ to detect potential data leakage. Although our benchmark has no direct overlap with public datasets, some medical knowledge (e.g., common procedures) may appear in LLM training corpora. For each prompt, we generated 50 outputs via nucleus sampling with temperature *t* = 0.8 and one greedy decoding, then computed token-level edit distances between each sample and the greedy output. We defined peakedness as the fraction of samples within an edit distance threshold α = 0.05 × length. A prompt was flagged as contaminated if its peakedness exceeded a threshold of 0.01. The overall contamination rate was calculated as the proportion of such prompts across the test set. Using this procedure, we identified clean and contaminated samples for the LLMs and further selected 200 uncontaminated samples to assess the impact of contamination on both reasoning and QA prediction performance.

### Efficiency analysis

For each evaluation approach, we recorded the evaluation time once per LLM and calculated the average runtime across all evaluated LLMs.

### Error detection of reasoning evaluation

We performed an error detection analysis to evaluate the alignment between the errors identified by LLM-w-Rationale and those annotated by experts. Expert assessments of rationale correctness served as the ground truth, while the model’s assessments were treated as predictions. Based on these, we calculated the numbers of true positives (TP), true negatives (TN), false positives (FP), and false negatives (FN). Subsequently, precision, recall, and F_1_-score were computed to quantify the accuracy of LLM-w-Rationale in detecting reasoning errors. The evaluation metrics are defined as:

7 $${Precision}=\frac{{TP}}{{TP}+{FP}}$$

8 $${Recall}=\frac{{TP}}{{TP}+{FN}}$$

9 $${F}_{1}=\frac{2\times {Precision}\times {Recall}}{{Precision}+{Recall}}$$

### Statistical analysis

A non-parametric bootstrap procedure with 1000 iterations was employed to estimate the mean and 95% confidence intervals of the evaluation metrics. In each iteration, a resampled dataset equal in size to the test set was generated via random sampling with replacement.

## Supplementary information

Supplementary Information

## Data Availability

The medical questions are sourced from public datasets, which may be available for research purposes upon reasonable request. 1. Medbullets: https://huggingface.co/datasets/LangAGI-Lab/medbullets, 2. MMLU-Pro: https://huggingface.co/datasets/TIGER-Lab/MMLU-Pro, 3. MedExQA: https://huggingface.co/datasets/bluesky333/MedExQA, 4. MedXpertQA: https://huggingface.co/datasets/TsinghuaC3I/MedXpertQA, 5. Humanity’s Last Exam: https://github.com/centerforaisafety/hle, 6. MedQA-USMLE: https://huggingface.co/datasets/bigbio/med_qa, 7. PubMedQA: https://huggingface.co/datasets/bigbio/pubmed_qa, 8. MedMCQA: https://huggingface.co/datasets/lighteval/med_mcqa, 9. MMLU-Medicine: https://huggingface.co/datasets/cais/mmlu, 10. HEAD-QA: https://huggingface.co/datasets/dvilares/head_qa. The expert-curated data with fine-grained rationale annotation is released at https://github.com/plusnli/MedThink-Bench.

## Code availability

The code is publicly available at https://github.com/plusnli/MedThink-Bench.
