# Supplementary material for: Automating expert-level medical reasoning evaluation of large language models
Source: NPJ Digit Med. 2025 Dec 6;9:34. doi: 10.1038/s41746-025-02208-7 (PMC12796170; doi:10.1038/s41746-025-02208-7)
Supplement: Supplementary file 1 — Supplementary Information [file 41746_2025_2208_MOESM1_ESM.pdf]

Supplementary Note 1

In this study, we utilized medical questions curated from ten publicly available datasets. Specifically, we primarily sourced the questions from the validation or test sets of the original datasets.

Supplementary Table 1. Data statistics of the source medical question datasets.

Index	Dataset	Data size
1	MedQA-USMLE	1273
2	PubMedQA	500
3	MedMCQA	2816
4	MMLU (Medical Subset)	1089
5	MedBullets	308
6	MMLU-Pro	818
7	MedExQA	935
8	MedXpertQA	2450
9	HEAD-QA	2472
10	Humanity's Last Exam (Medicine)	275

Supplementary Note 2

This section outlines the data preprocessing steps applied to the collected public datasets. After the acquisition, we first removed duplicate questions, where applicable. Given that some questions required accompanying medical images for proper interpretation, yet images were not available, we excluded these questions using a keyword-based filtering approach.

Subsequently, we identified complex medical questions through a structured annotation process conducted by our expert team. We define a complex question as one that requires at least two intermediate reasoning steps to reach a clinically valid and contextually appropriate answer. These questions go beyond surface-level factual recall and require higher-order cognitive processes, such as synthesis, abstraction, and multi-step deduction. In contrast, simple questions typically require only a single-step response based on direct retrieval of medical knowledge.

Each question was independently reviewed by two annotators. In cases of disagreement, the annotators engaged in discussion to reconcile differences and reach a consensus. To assess annotation reliability, we computed the inter-annotator agreement (IAA) using Cohen's Kappa (κ). Specifically, we calculated κ by alternately treating each annotator's labels as the ground truth and averaging the two resulting scores. The final κ value for complex question identification was 0.89, reflecting a high level of agreement and annotation consistency.

Supplementary Note 3

Guideline of Expert Annotation

Definition of Ten Medical Domains

Diagnostic Workup: This domain encompasses the processes involved in collecting and synthesizing patient information to support clinical reasoning. It includes history taking, physical examination, laboratory investigations, imaging, and pathology tests. Unlike *Disease Diagnosis*, which centers on reaching a final clinical conclusion, *Diagnostic Workup* is focused on information gathering and identifying relevant clinical signs to guide further evaluation.

Disease Diagnosis: This domain involves the integration of clinical findings, test results, and patient presentation to identify the most likely disease or condition. It is centered on diagnostic reasoning and decision-making. In contrast to *Pathology*, which emphasizes molecular and cellular mechanisms of disease, *Disease Diagnosis* focuses on clinical-level interpretation rather than mechanistic explanations.

Treatment: This domain covers the selection and application of therapeutic interventions aimed at managing or curing disease. It includes pharmacologic treatments, surgical procedures, etc. It is distinct from *Pharmacology*, which delves into drug mechanisms and properties, and from *Discharge*, which focuses on post-treatment care.

Prognosis: This domain addresses the anticipated course and outcomes of a disease, including recovery potential, disease progression, recurrence risks, and long-term health impacts. It differs from *Treatment*, which involves active clinical interventions, and from *Discharge*, which deals with short-term care planning.

Discharge: This domain focuses on the transition from inpatient to outpatient care, including discharge planning, patient education, follow-up scheduling, and long-term management strategies.

Pharmacology: This domain involves the study of drug actions, indications, contraindications, mechanisms of action, side effects, and drug interactions. It is mechanistic and therapeutic in nature, differing from *Treatment*, which concerns the application of those drugs (or other interventions) in a clinical context.

Anatomy & Physiology: This domain covers the normal structure and function of the human body at both macro and micro levels. It provides the foundational knowledge required to understand disease processes. Unlike *Pathology*, which explores abnormal functions and structural changes due to disease, *Anatomy & Physiology* focuses exclusively on *normal biological function and structure*.

Pathology: This domain examines the biological and physiological mechanisms underlying disease processes, including changes at the molecular and cellular levels. It provides

mechanistic insights into disease development and differs from *Disease Diagnosis*, which uses clinical reasoning to identify conditions, and from *Anatomy & Physiology*, which describes normal states.

Public Health: This domain focuses on population-level health issues, including disease prevention, health promotion, epidemiology, and the identification and management of risk factors, whether behavioral, biological, or environmental. It stands apart from *Clinical Treatment* domains by emphasizing prevention over cure and from *Policy & Ethics* by prioritizing scientific and public health principles over regulatory or ethical dimensions.

Policy & Ethics: This domain encompasses healthcare regulations, ethical dilemmas, and legal responsibilities in medical practice. It involves decisions regarding patient autonomy, resource allocation, consent, and professional conduct. Unlike *Public Health*, which focuses on epidemiological and preventive strategies, *Policy & Ethics* examines *value-laden decisions* and *regulatory frameworks* that shape healthcare delivery.

Question Type Annotation

Following the identification of complex questions involving multi-step reasoning, we performed question type annotation. Each medical question was manually classified into one of ten predefined medical domains. Annotation was carried out independently by two annotators. In cases of disagreement, the annotators discussed their classifications to reach a consensus. If consensus could not be achieved, a third annotator was consulted to make the final determination. To assess the reliability of the annotation process, we calculated inter-annotator agreement (IAA) using Cohen's Kappa (κ). The final κ score for the question type annotation was 0.81, indicating a high level of consistency.
The annotation was conducted by a team comprising three senior and seven junior annotators. The senior members (C.E., L.W., and E.S.) are licensed physicians holding medical degrees. Among the junior annotators, three (Y.C., X.M., and M.T.) are medical students, one (Y.X.) is from a dental program, and three others (M.S., Y.J., and Z.X.) are enrolled in a nursing program.

Data Type Annotation Examples

Case 1:
Question: A 22-year-old female patient seeks medical attention for back pain following a softball incident from the previous day. While sliding headfirst to home plate, she experienced a "twinge" in her lower back. She reports no numbness, tingling, or radiating pain. Physical examination reveals paravertebral tenderness from L2–L4, with the physician's right thumb positioned more anteriorly than the left when palpating the transverse processes along these vertebrae. Forward bending increases tenderness, but palpatory findings remain unchanged. The physician plans to treat using a technique involving engagement of the restrictive barrier followed by a brief corrective impulse. What is the most appropriate positioning for treating this patient's somatic dysfunction?

Answer Choices: (A) The seated position with forward flexion and arms crossed (B) The right lateral recumbent position with both arms crossed over chest (C) The prone position with arms extended overhead (D) The left lateral recumbent position and the right upper extremity pulled inferiorly (E) The left lateral recumbent position and the left upper extremity pulled inferiorly (F) The left lateral recumbent position and the left upper extremity pulled superiorly (G) The prone position with bilateral upper extremities at sides (H) The right lateral recumbent position and the left upper extremity pulled superiorly (I) The right lateral recumbent position and the right upper extremity pulled inferiorly (J) The supine position with knees flexed and arms at sides

Type: Treatment

Case 2:
Question: A 37-year-old machinist presents to his primary care physician with eye problems. He has felt a mass in his eye that has persisted for the past month. The patient has a medical history of blepharitis treated with eye cleansing and squamous cell carcinoma of the skin treated with Mohs surgery. His temperature is 99.5°F (37.5°C), blood pressure is 157/102 mmHg, pulse is 90/min, respirations are 17/min, and oxygen saturation is 98% on room air. Physical exam is notable for a firm and rubbery nodule palpable inside the patient's left eyelid. Physical exam does not elicit any pain. Which of the following is the most likely diagnosis?
A. Chalazion
B. Foreign body
C. Hordeolum
D. Meibomian cell carcinoma

Type: Diagnosis

Case 3:
Question: A 67-year-old male is seen by neurology after he was noticed to be speaking strangely by his family. After acute treatment with tissue plasminogen activator (tPA), the patient is able to recover most of his speech. Subsequent neurologic exam finds that the patient is fluent while speaking and is able to comprehend both one and two step instructions. Noticeably the patient remains unable to complete tasks involving verbal repetition. Residual damage to which of the following structures is most likely responsible for this patient's syndrome?
A. Arcuate fasciculus
B. Inferior frontal gyrus
C. Superior temporal gyrus
D. Inferior frontal gyrus + superior temporal gyrus
E. Arcuate fasciculus + inferior frontal gyrus + superior temporal gyrus

Type: Anatomy & Physiology

Case 4:

Question: A 5-year-old boy is brought to the emergency department with complaints of a sore throat and difficulty breathing. His mother reports that the symptoms began the previous night and have worsened rapidly. The child is normally healthy, up to date on all childhood vaccinations, and takes a daily multivitamin. His temperature is 103°F (39.4°C), blood pressure is 100/64 mmHg, pulse is 155/min, respirations are 29/min, and oxygen saturation is 95% on room air. On examination, he appears ill, is drooling, and leans forward to breathe. He is nonverbal, visibly uncomfortable, and does not cooperate with an oral examination. What is the most likely infectious cause of this patient's symptoms?
Answer Choices: (A) Haemophilus influenzae (B) Streptococcus pneumoniae (C) Parainfluenza virus (D) Staphylococcus aureus (E) Group A Streptococcus (F) Adenovirus (G) Mycoplasma pneumoniae (H) Epstein-Barr virus (I) Corynebacterium diphtheriae (J) Respiratory syncytial virus (RSV)

Type: Public Health

Case 5:
Question: A 30-year-old woman comes to the clinic with a complaint of itchy red fluid-filled rashes over the left forearm for the last two days. She is applying neomycin sulfate for a boil in that area for the past few days. On physical examination, erythematous pruritic vesicular eruptions are seen in the field of neomycin application over the left forearm. Which of the following cells is primarily responsible for the development of these rashes?
Answer Choices: (A) Natural killer (NK) cell (B) Memory T cell (C) Cytotoxic T cell (D) Plasma cell (E) Langerhans cell (F) Helper T cell (G) Dendritic cell (H) Eosinophil (I) Neutrophil (J) Mast cell

Type: Pathology

Reasoning Annotation

The core objective of reasoning annotation is to provide fine-grained, step-by-step reasoning trajectories that accurately reflect real-world clinical logic. For each annotation task, annotators were given the clinical question along with the ground-truth answer and asked to decompose the reasoning process into discrete, logically coherent steps that collectively lead to the correct answer.

To ensure that the reasoning trajectories effectively evaluate a model's reasoning capability, we established three key requirements. First, only essential reasoning steps were included, ensuring that each step was indispensable for arriving at the answer and thereby enhancing the necessity of the annotations. Second, annotators were instructed not to omit any critical steps, promoting completeness and capturing the full breadth of clinical reasoning. Third, each step had to be explicitly supported by evidence from the question, reinforcing the cohesiveness of the annotation and ensuring that all reasoning was grounded in the provided clinical context.

Importantly, annotators were prohibited from using a process-of-elimination strategy that merely discards incorrect options to justify the correct answer. Instead, annotations were required to follow constructive, evidence-based reasoning. Annotators were permitted to consult external

resources (e.g., Internet, medical literature, or guidelines) during the annotation process. To ensure consistency, all annotators received standardized training, including written instructions and exemplar annotations.

Annotation was conducted iteratively. Initially, two annotators independently labeled a set of 10 cases, after which they discussed discrepancies and calculated inter-annotator agreement (IAA). If disagreements could not be resolved through discussion, a third physician reviewed the case and provided the final annotation. The annotators then repeated this process with 20 additional cases, followed by another batch of 30 cases, each time refining their approach to improve consistency. This iterative cycle was repeated until the entire dataset was annotated, resulting in a high-quality gold standard dataset.

We quantified IAA for reasoning annotation using Cohen's Kappa (κ). For each reasoning step, agreement between two annotators was scored as 1 if the main semantic content was consistent and 0 if it diverged. Since each annotation consisted of multiple reasoning steps, agreement was computed at the entry level and then aggregated. The final κ score for reasoning annotation reached 0.73, indicating a high level of consistency across annotators.

Reasoning Annotation Examples

Case 6:

Question: A 62-year-old patient has been hospitalized for a week due to a stroke. One week into the hospitalization, he develops a fever and purulent cough. His vitals include: heart rate 88/min, respiratory rate 20/min, temperature 38.4°C (101.1°F), and blood pressure 110/85 mm Hg. On physical examination, he has basal crackles on the right side of the chest. Chest radiography shows a new consolidation on the same side. Complete blood count is as follows: Hemoglobin 16 mg/dL, Hematocrit 50%, Leukocyte count 8,900/mm3, Neutrophils 72%, Bands 4%, Eosinophils 2%, Basophils 0%, Lymphocytes 17%, Monocytes 5%, Platelet count 280,000/mm3.

What is the most likely causal microorganism?

A. Pseudomona aeruginosa

B. Streptococcus pneumoniae

C. Mycobacterium tuberculosis

D. Haemophilus influenzae

E. Staphylococcus aureus

Answer: E. Staphylococcus aureus

Reasoning Annotation:

['Laboratory Results: leukocyte count 8,900/mm^3, Neutrophils 72%, together with the high body temperature 38.4°C (101.1°F), suggests a bacterial infection',

'A hospitalized patient who develops new fever, purulent cough, and consolidation on chest X-ray one week after admission is suspicious for hospital-acquired pneumonia (HAP)',

'For non-ventilated early-onset HAP patients, Staphylococcus aureus is the most common pathogen']

Case 7:

Question: A 27-year-old woman presents to her primary care physician with pain in her hands, shoulders, and knees. The pain has lasted for several months but seems to have worsened recently. Any activity such as opening jars, walking, or brushing her teeth is painful. The patient has a medical history of a suicide attempt in college, constipation, anxiety, depression, and a sunburn associated with surfing which was treated with aloe vera gel. Her temperature is 99.5°F (37.5°C), blood pressure is 137/78 mmHg, pulse is 92/min, respirations are 14/min, and oxygen saturation is 98% on room air. Laboratory values are obtained and shown below. Hemoglobin: 9 g/dL Hematocrit: 33% Leukocyte count: 2,500/mm^3 with normal differential Platelet count: 107,000/mm^3 Serum: Na+: 139 mEq/L Cl-: 102 mEq/L K+: 4.4 mEq/L HCO3-: 24 mEq/L BUN: 21 mg/dL Glucose: 90 mg/dL Creatinine: 1.0 mg/dL Ca2+: 10.2 mg/dL AST: 12 U/L ALT: 10 U/L Which of the following findings is the most likely to be positive in this patient?
A. Anti-cyclic citrullinated peptide antibodies
B. Anti-dsDNA antibodies
C. Anti-histone antibodies
D. Degenerated cartilage in weight bearing joints
E. IgM against parvovirus B19

Answer:
B. Anti-dsDNA antibodies

Reasoning Annotation:
['This female patient presents with chronic polyarticular pain, possible photosensitivity (sunburn history), depression history, and pancytopenia (anemia, leukopenia, thrombocytopenia), which are classic features of systemic lupus erythematosus.',
'Anti-dsDNA antibodies are highly specific for SLE and represent the most likely positive finding.']

Case 8:

Question: A 65-year-old woman presents with pain, swelling, and warmth in her right knee 18 hours after receiving a methylprednisolone-lidocaine injection for chronic osteoarthritis in that knee. Her symptoms began approximately 6 hours post-injection and have not improved. She denies fever, chills, or other systemic symptoms and is otherwise healthy.
On examination, she is afebrile with normal vital signs. There is increased tenderness over the medial and lateral joint lines compared to before the injection, along with a new moderate knee effusion. Her range of motion is limited in both flexion and extension, with pain on passive movement. Ligamentous testing shows no laxity.
Joint aspiration yields 25 mL of cloudy yellow fluid. Laboratory analysis of the fluid reveals 12,000 leukocytes/mm³ with 95% polymorphonuclear cells. Gram stain is negative. Crystal analysis using compensated polarized light microscopy shows abundant intra- and extracellular crystals with polymorphic appearance and both positive and negative birefringence.
What is the most appropriate next step in managing this patient?
Answer Choices: (A) Initiate treatment with colchicine (B) Perform repeat joint aspiration to confirm the diagnosis (C) Withhold treatment and observe for spontaneous resolution (D) Arrange for orthopedic arthroscopic lavage of the knee (E) Apply ice, and initiate treatment with ibuprofen (F) Initiate treatment with allopurinol for chronic gout (G) Prescribe antibiotics for

presumed septic arthritis (H) Start oral prednisone and monitor for improvement (I) Refer for emergent orthopedic surgery consultation (J) Administer intra-articular corticosteroids

Answer:
E. Apply ice, and initiate treatment with ibuprofen

Reasoning Annotation:
['The patient is a 65-year-old woman experiencing acute right knee pain, swelling, and warmth 6 hours after corticosteroid injection, raising concern for a post-injection complication.',
'Synovial fluid analysis shows 12,000 leukocytes/mm³ with 95% neutrophils and abundant crystals with both positive and negative birefringence, indicating mixed crystal arthritis.',
'The absence of fever, negative Gram stain, and timing shortly after steroid injection make crystal-induced arthritis more likely than septic arthritis.',
'The most appropriate next step in management is to apply ice and initiate NSAID therapy, such as ibuprofen, to reduce inflammation and relieve symptoms.']

Supplementary Note 4

In the experiment, we employed a zero-shot Chain-of-Thought (CoT) prompting method to facilitate LLMs in generating medical reasoning during inference.

Prompt:
You are an experienced doctor. Your task is to answer the medical question below.

Question:
{question}

Let's think about this question step by step, and you should provide the detailed reasoning process.
(Please write your reasoning process here.)

Based on the above reasoning analysis, please give the final answer here.
(Notably, the final answer should be quoted with Square Brackets. For example, ["A. option1"], ["B. option2"], etc.)

Supplementary Note 5

In the human evaluation, each medical expert was provided with the medical question, the corresponding answer, the model-generated rationale, and a detailed grading scheme to assess the quality of the predicted rationale. The grading scheme served as a standardized protocol to ensure consistency and uniformity throughout the evaluation process.
Experts examined each rationale step by step, recording (1) the number of reasoning steps they considered necessary to reach the correct answer for that question, and (2) how many of those necessary steps were present in the model's rationale. Each question was independently reviewed by two experts, who were allowed to consult external resources (e.g., Internet, medical literature, or guidelines) during the grading process. In cases of disagreement, the reviewers discussed the discrepancies and reached a consensus.

Grading Scheme:
Based on the provided materials, please first determine the number of reasoning steps you considered necessary to reach the correct answer and then judge how many of those essential steps were present in the model's rationale.

When determining the necessary reasoning steps (ground-truth rationales), three general principles should be followed:
First, include only essential reasoning steps, ensuring that each step is indispensable for arriving at the answer.
Second, do not omit any critical steps, as this promotes completeness and captures the full scope of clinical reasoning.
Third, ensure that each step is explicitly supported by evidence from the question.

When assessing how many of these essential steps (ground-truth rationales) are present in the model's rationale, please follow the following point-wise grading approach:
- A ground-truth rationale is considered mentioned only if **its main content** is **explicitly stated** in the models prediction.
- **Do not award the statement** if any key information of the ground-truth rationale is missing from the models prediction.
- **Do not award the statement** if any information in the models prediction conflicts with the ground-truth rationale.
- **Do not award the statement** if only a small part of the ground-truth rationale is mentioned in the models prediction.

Supplementary Note 6

This section introduces the prompts used for Reference-based LLM-as-a-Judge (LLM-w-Rationale) and Reference-free LLM-as-a-Judge (LLM-w/o-Rationale) to assess the quality of model-generated rationales.

Prompts for LLM-w-Rationale:

You are a meticulous medical QA grader.
Given a question, a ground-truth rationale, and a model's prediction (which may include reasoning), evaluate whether the prediction comprehensively mentions the ground-truth rationale.
Grade in a point-wise manner:
*- The ground-truth rationale is considered mentioned only if **its main content** is **explicitly stated** in the models prediction.*
*- **Do not award the statement** if any key information of the ground-truth rationale is missing from the models prediction.*
*- **Do not award the statement** if any information in the models prediction conflicts with the ground-truth rationale.*
*- **Do not award the statement** if only a small part of the ground-truth rationale is mentioned in the models prediction.*

Finally, please output a Boolean value to show whether the prediction mentions the ground-truth rationale.

Prompts for LLM-w/o-Rationale:

You are a meticulous medical QA grader.

Given a question and its answer, you should first generate a list of nuanced reasoning steps that can accurately lead to the correct answer. Then, you should take the generated reasoning steps as ground-truth rationale to assess the quality of model-generated rationales from a prediction model.

Specifically, given the question and a model's prediction (which may include reasoning), evaluate whether the prediction comprehensively mentions your generated rationale (i.e., ground-truth rationale).
Grade in a point-wise manner:
*- The ground-truth rationale is considered mentioned only if **its main content** is **explicitly stated** in the models prediction.*
*- **Do not award the statement** if any key information of the ground-truth rationale is missing from the models prediction.*
*- **Do not award the statement** if any information in the models prediction conflicts with the ground-truth rationale.*

- **Do not award the statement** if only a small part of the ground-truth rationale is mentioned in the models prediction.

Finally, please output the total number of reasoning steps in the ground-truth rationale (an integer) and the number of the recalled reasoning steps (an integer).

Supplementary Note 7

We compared the prediction accuracy of the investigated LLMs on the MedThink-Bench.

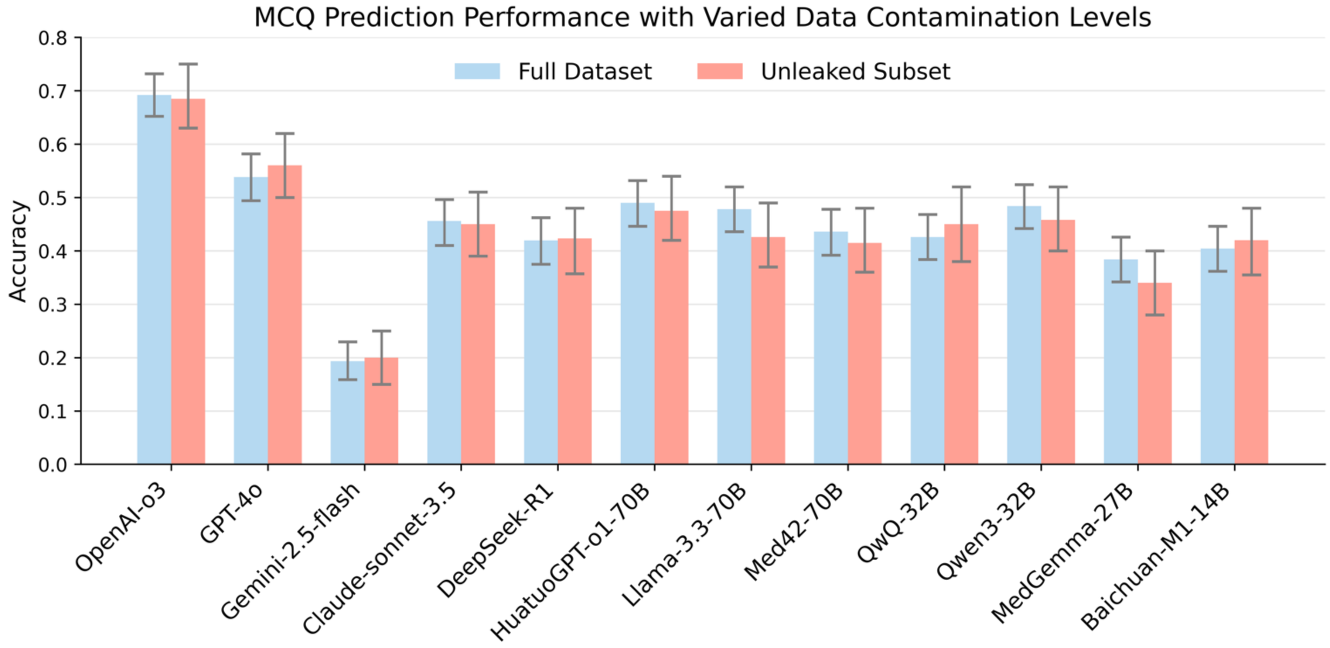

Supplementary Figure 1. Prediction performance on the full dataset (blue bars) and an uncontaminated subset (red bars) of MedThink-Bench. Experiments were conducted under zero-shot prompting. The assessments were obtained by comparing ground-truth answers with the predicted ones. Error bars represent the 95% CI of the mean, calculated via bootstrapping.

Supplementary Note 8

To analyze the correlation between expert and automated evaluation, we further visualized the individual evaluation scores of OpenAI-o3, HuatuoGPT-o1-70B, and Baichuan-M1-14B.

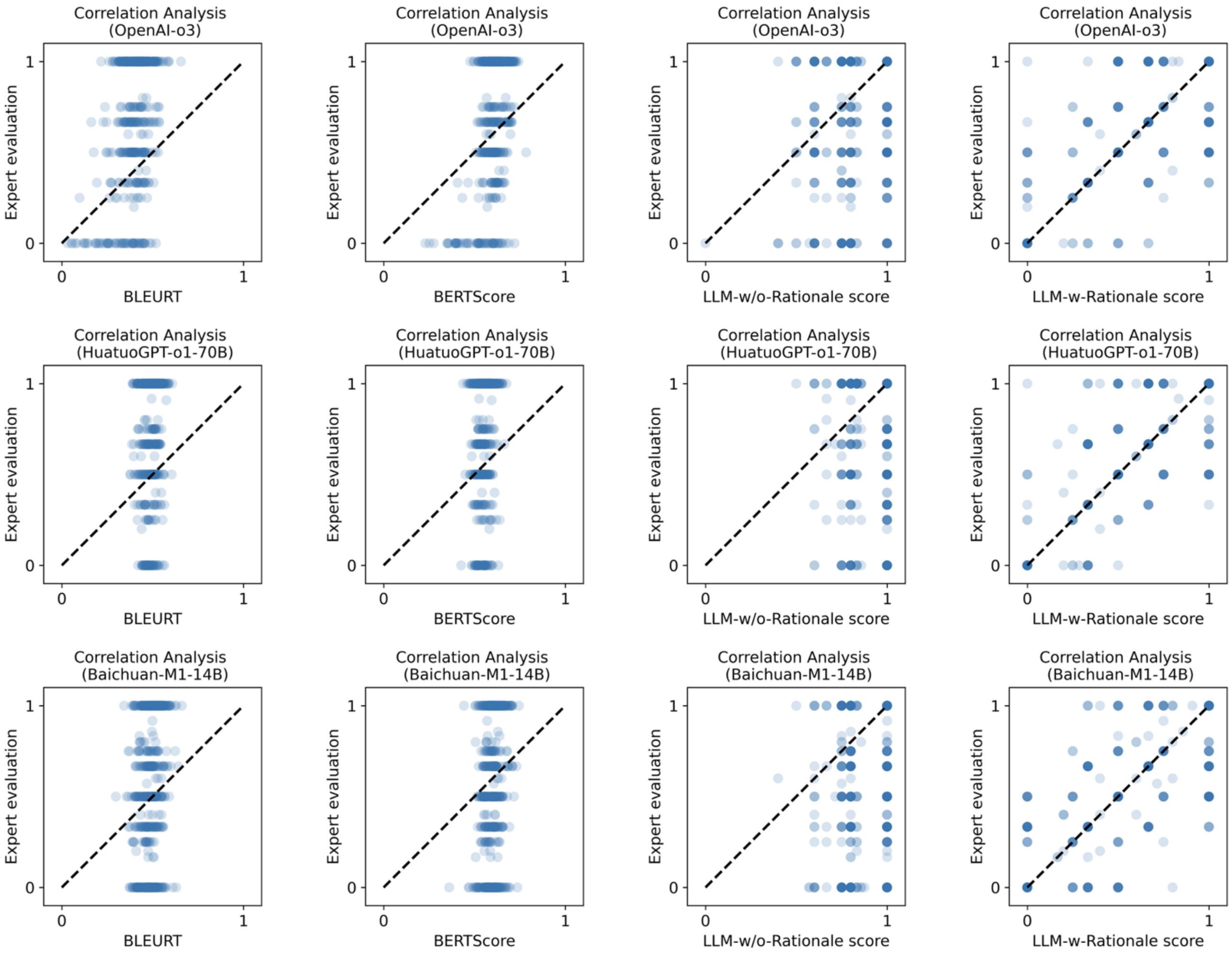

Supplementary Figure 2. Scatter plot illustrating the correlation between expert evaluation scores and automated evaluation scores for individual samples in the MedThink-Bench dataset. The plot compares results across three models: OpenAI-o3, HuatuoGPT-o1-70B, and Baichuan-M1-14B. Automated evaluation was conducted using two LLM-based metrics (LLM-w-Rationale and LLM-w/o-Rationale), where GPT-4o-mini served as the judge model. Traditional metrics (BLEURT and BERTScore) were also included for comparison. Each point represents a single sample, with dashed lines denoting perfect agreement between expert and automated scores. Notably, LLM-w-Rationale shows strong alignment with expert evaluations, as evidenced by points clustering near the dashed line. In contrast, BLEURT, BERTScore, and LLM-w/o-Rationale demonstrate greater deviation, reflecting weaker concordance with expert judgment.

Supplementary Note 9

We investigated the sensitivity of the LLM-w-Rationale framework to different prompt formulations by composing five prompt variations with similar semantics. We used the first prompt for most of the experiments in the study.

Prompt 1:

You are a meticulous medical QA grader.
Given a question, a ground-truth rationale, and a model’s prediction (which may include reasoning), evaluate whether the prediction comprehensively mentions the ground-truth rationale.
Grade in a point-wise manner:
- The ground-truth rationale is considered mentioned only if **its main content** is **explicitly stated** in the models prediction.
- **Do not award the statement** if any key information of the ground-truth rationale is missing from the models prediction.
- **Do not award the statement** if any information in the models prediction conflicts with the ground-truth rationale.
- **Do not award the statement** if only a small part of the ground-truth rationale is mentioned in the models prediction.

Prompt 2:

You are a meticulous medical QA grader.
Given a question, a ground-truth rationale, and a model’s prediction (which may include reasoning), evaluate whether the prediction comprehensively mentions the ground-truth rationale.
Grade in a point-wise manner:
- The ground-truth rationale is acknowledged as mentioned only when its primary content is **clearly articulated** in the model’s response.
- No credit should be given if the model’s prediction **omits any critical detail** of the ground-truth rationale.
- No credit should be given if the model’s prediction **includes any content that contradicts** the ground-truth rationale.
- No credit should be given if the model’s prediction **covers only a minor portion** of the ground-truth rationale.

Prompt 3:

You are a meticulous medical QA grader.
Given a question, a ground-truth rationale, and a model’s prediction (which may include reasoning), evaluate whether the prediction comprehensively mentions the ground-truth rationale.
Grade in a point-wise manner:

- A model's prediction is deemed to align with the ground-truth rationale only when the core content is **clearly and explicitly conveyed**.
- No credit should be assigned if **essential details from the ground-truth rationale are omitted** in the prediction.
- No credit should be assigned if the prediction contains elements that **contradict the ground-truth rationale**.
- No credit should be given if the model's response **includes merely a minor aspect** of the ground-truth rationale.

Prompt 4:

You are a meticulous medical QA grader.
Given a question, a ground-truth rationale, and a model's prediction (which may include reasoning), evaluate whether the prediction comprehensively mentions the ground-truth rationale.
Grade in a point-wise manner:
- The model's prediction must **explicitly include the core content** of the ground-truth rationale to be considered a match.
- A prediction must **fully cover the key elements** of the ground-truth rationale, i.e., **omission of key elements results in disqualification**.
- **Any contradiction** between the prediction and the ground-truth rationale leads to rejection.
- No credit is given if **only minor or tangential elements** of the ground-truth rationale are referenced without capturing its main points.

Prompt 5:

You are a meticulous medical QA grader.
Given a question, a ground-truth rationale, and a model's prediction (which may include reasoning), evaluate whether the prediction comprehensively mentions the ground-truth rationale.
Grade in a point-wise manner:
- The ground-truth rationale is accepted as identified **only when its essential content is clearly represented** in the model's response.
- No points should be awarded if the model's prediction **fails to include any essential aspect** of the ground-truth rationale.
- No points should be awarded if the model's prediction **contains any elements that conflict with** the ground-truth rationale.
- No points should be awarded if the model's prediction addresses **merely a small segment** of the ground-truth rationale.

Supplementary Note 10

We evaluated the robustness of LLM-w-Rationale to different judge models. We used the rationales predicted by OpenAI-o3, Qwen3-32B, and MedGemma-27B and tested ten different LLMs as the judge model.

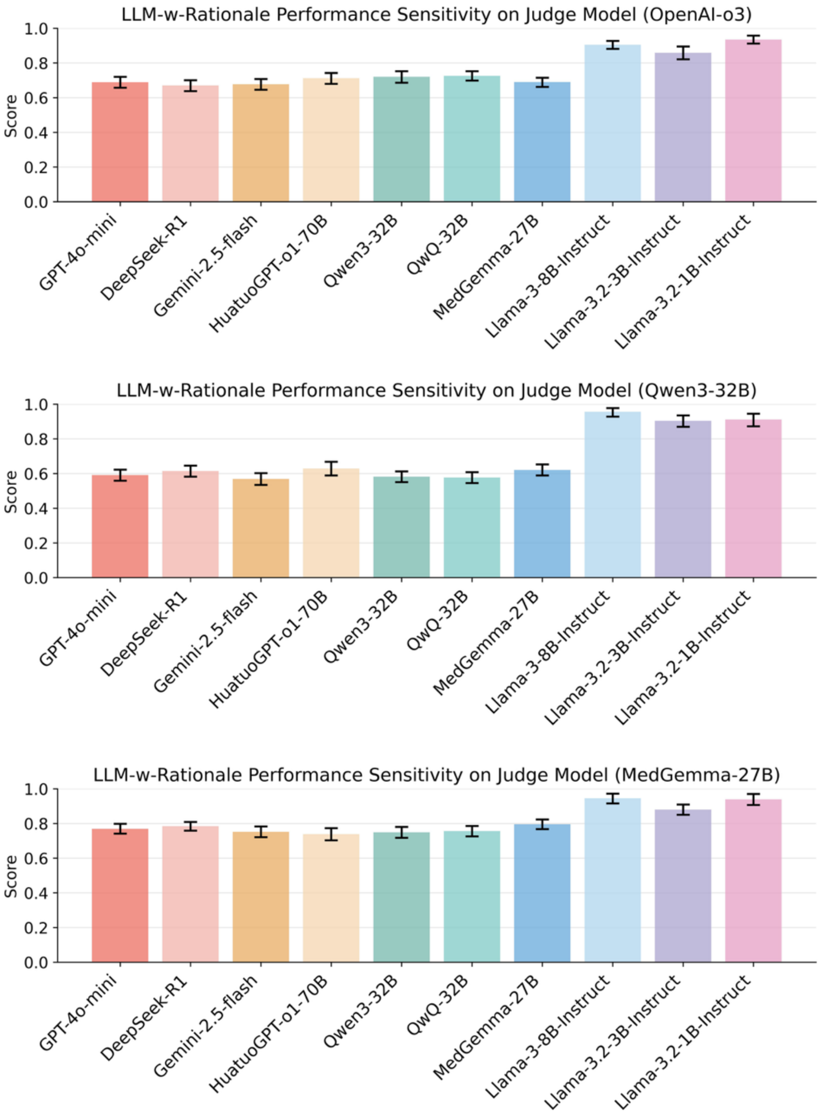

Supplementary Figure 3. Robustness of LLM-w-Rationale across different judge models. Predicted rationales from OpenAI-o3, Qwen3-32B, and MedGemma-27B were evaluated using ten LLMs of varying scales as judge models. Error bars represent the 95% CI of the mean, calculated via bootstrapping. The evaluation outcomes remain stable when using judge models with strong instruction-following capabilities, such as GPT-4o-mini or Qwen3-32B.

Supplementary Note 11

To illustrate the effectiveness of the LLM-w-Rationale metric in evaluating the medical reasoning capabilities of LLMs, we conducted a series of case studies. Three representative examples are presented. The first example demonstrates a fully correct reasoning process leading to the correct answer. In contrast, the second and third examples reveal flawed or incomplete reasoning pathways, despite ultimately arriving at the correct prediction. These cases highlight the ability of LLM-w-Rationale to distinguish not only between correct and incorrect answers but also to offer a more nuanced evaluation of an LLM's medical reasoning capabilities.

Type: Disease diagnosis

Question: A 27-year-old woman presents to her primary care physician with pain in her hands, shoulders, and knees. The pain has lasted for several months but seems to have worsened recently. Any activity such as opening jars, walking, or brushing her teeth is painful. The patient has a medical history of a suicide attempt in college, constipation, anxiety, depression, and a sunburn associated with surfing which was treated with aloe vera gel. Her temperature is 99.5°F (37.5°C), blood pressure is 137/78 mmHg, pulse is 92/min, respirations are 14/min, and oxygen saturation is 98% on room air. Laboratory values are obtained and shown below. Hemoglobin: 9 g/dL Hematocrit: 33% Leukocyte count: 2,500/mm^3 with normal differential Platelet count: 107,000/mm^3 Serum: Na^+: 139 mEq/L Cl^-: 102 mEq/L K^+: 4.4 mEq/L HCO_3^-: 24 mEq/L BUN: 21 mg/dL Glucose: 90 mg/dL Creatinine: 1.0 mg/dL Ca^{2+}: 10.2 mg/dL AST: 12 U/L ALT: 10 U/L Which of the following findings is the most likely to be positive in this patient?

A. Anti-cyclic citrullinated peptide antibodies B. Anti-dsDNA antibodies C. Anti-histone antibodies D. Degenerated cartilage in weight bearing joints E. IgM against parvovirus B19

Ground-truth answer: B. Anti-dsDNA antibodies.

Predicted answer: B. Anti-dsDNA antibodies.

Fine-grained reasoning annotation:

- This female patient presents with chronic polyarticular pain, possible photosensitivity (sunburn history), depression history, and pancytopenia (anemia, leukopenia, thrombocytopenia), which are classic features of systemic lupus erythematosus
- Anti-dsDNA antibodies are highly specific for SLE and represent the most likely positive finding.

Predicted rationale:

The patient's symptoms include pain in her hands, shoulders, and knees, which has worsened recently. She also has a history of constipation, anxiety, depression, and a sunburn. The laboratory values show anemia (hemoglobin: 9 g/dL, hematocrit: 33%), leukocytosis (leukocyte count: 2,500/mm^3), and thrombocytosis (platelet count: 107,000/mm^3). The electrolytes and kidney function are normal. The patient's symptoms and laboratory findings are suggestive of an autoimmune disorder.

The patient's symptoms and laboratory findings are most consistent with systemic lupus erythematosus (SLE), which is an autoimmune disorder characterized by the presence of anti-dsDNA antibodies.

Reasoning correctness: 1.0

Supplementary Figure 4. Case study of medical reasoning assessment. This case demonstrates that the prediction model Llama-3.3-70B produced a correct answer and generated correct reasoning trajectories (highlighted in red).

Type: Treatment

Question: A 65-year-old woman presents with pain, swelling, and warmth in her right knee 18 hours after receiving a methylprednisolone-lidocaine injection for chronic osteoarthritis in that knee. Her symptoms began approximately 6 hours post-injection and have not improved. She denies fever, chills, or other systemic symptoms and is otherwise healthy. On examination, she is afebrile with normal vital signs. There is increased tenderness over the medial and lateral joint lines compared to before the injection, along with a new moderate knee effusion. Her range of motion is limited in both flexion and extension, with pain on passive movement. Ligamentous testing shows no laxity. Joint aspiration yields 25 mL of cloudy yellow fluid. Laboratory analysis of the fluid reveals 12,000 leukocytes/mm³ with 95% polymorphonuclear cells. Gram stain is negative. Crystal analysis using compensated polarized light microscopy shows abundant intra- and extracellular crystals with polymorphic appearance and both positive and negative birefringence. What is the most appropriate next step in managing this patient?

Answer Choices: (A) Initiate treatment with colchicine (B) Perform repeat joint aspiration to confirm the diagnosis (C) Withhold treatment and observe for spontaneous resolution (D) Arrange for orthopedic arthroscopic lavage of the knee (E) Apply ice, and initiate treatment with ibuprofen (F) Initiate treatment with allopurinol for chronic gout (G) Prescribe antibiotics for presumed septic arthritis (H) Start oral prednisone and monitor for improvement (I) Refer for emergent orthopedic surgery consultation (J) Administer intra-articular corticosteroids

---

Ground-truth answer: E. Apply ice, and initiate treatment with ibuprofen.

Predicted answer: E. Apply ice, and initiate treatment with ibuprofen.

Fine-grained reasoning annotation:

- The patient is a 65-year-old woman experiencing acute right knee pain, swelling, and warmth 6 hours after corticosteroid injection, raising concern for a post-injection complication.
- Synovial fluid analysis shows 12,000 leukocytes/mm³ with 95% neutrophils and abundant crystals with both positive and negative birefringence, indicating mixed crystal arthritis.
- The absence of fever, negative Gram stain, and timing shortly after steroid injection make crystal-induced arthritis more likely than septic arthritis.
- The most appropriate next step in management is to apply ice and initiate NSAID therapy, such as ibuprofen, to reduce inflammation and relieve symptoms.

Predicted rationale:

1. Patient profile: 65-year-old woman with osteoarthritis who recently received a steroid injection in her knee. 2. Presenting symptoms: Pain, swelling, and warmth in the injected knee, starting 6 hours post-injection. 3. Physical examination: Shows increased tenderness, new moderate knee effusion, and limited range of motion 4. Joint aspiration results: - 25 mL of cloudy yellow fluid - 12,000 leukocytes/mm³ with 95% polymorphonuclear cells - Negative Gram stain - Crystal analysis shows abundant intra- and extracellular crystals with both positive and negative birefringence.

5. Key points: - The symptoms started shortly after the injection - The presence of crystals with both positive and negative birefringence suggests pseudogout (calcium pyrophosphate deposition disease) - The high leukocyte count and predominance of polymorphonuclear cells indicate an acute inflammatory response.

Management considerations:

- The condition is likely an acute pseudogout attack triggered by the steroid injection - Treatment should focus on reducing inflammation and managing pain - Antibiotics are not necessary as septic arthritis is less likely Based on this analysis, the most appropriate next step would be to treat the acute pseudogout attack with anti-inflammatory medication.

The best option from the given choices is: E. Apply ice, and initiate treatment with ibuprofen This approach will help reduce inflammation and manage pain. Ibuprofen, as a nonsteroidal anti-inflammatory drug (NSAID), is an appropriate first-line treatment for acute pseudogout attacks. Applying ice will also help with pain and swelling.

Reasoning correctness: 1/4

Supplementary Figure 5. Case study of medical reasoning assessment. This case demonstrates that the prediction model Claude-sonnet-3.5 produced a correct answer with flawed reasoning trajectories. The correct rationale is highlighted in red. It shows that LLM-w-Rationale can capture flawed reasoning patterns and offers a more nuanced evaluation of an LLM's medical capabilities.

Type: Treatment

Question: A 42-year-old female with a history of obesity, diabetes, and depression arrives at the emergency department with abdominal pain that began during dinner the previous night. Her vital signs show: temperature 104°F (40°C), blood pressure 90/65 mmHg, pulse 160/min, respirations 14/min, and oxygen saturation 98% on room air. The patient appears severely ill, with jaundiced skin, scleral icterus, and right upper quadrant tenderness. Ultrasound reveals a thickened, distended common bile duct. Laboratory results are as follows: - Hemoglobin: 13 g/dL - Hematocrit: 38% - Leukocyte count: 22,500 cells/mm^3 with normal differential - Platelet count: 257,000/mm^3 - Alkaline phosphatase: 355 U/L - Bilirubin, total: 11.3 mg/dL - Bilirubin, direct: 9.8 mg/dL - AST: 42 U/L - ALT: 31 U/L After initiating antibiotics and IV fluids, what is the most appropriate next step in management?

Answer Choices: (A) Endoscopic retrograde cholangiopancreatography (B) Exploratory laparotomy (C) Observation and supportive care (D) Magnetic resonance cholangiopancreatography (E) CT abdomen pelvis with contrast (F) Percutaneous transhepatic cholangiography (G) Repeat ultrasound with Doppler imaging (H) Hepatobiliary iminodiacetic acid (HIDA) scan (I) Antibiotic therapy alone (J) Surgical cholecystectomy without further imaging

---

Ground-truth answer: A. Endoscopic retrograde cholangiopancreatography.

Predicted answer: A. Endoscopic retrograde cholangiopancreatography.

Fine-grained reasoning annotation:

- The patient presents with Charcot's triad of fever, jaundice, and right upper quadrant pain along with hypotension and tachycardia, indicating acute suppurative cholangitis with early septic shock.
- Laboratory findings of leukocytosis, elevated alkaline phosphatase, and direct hyperbilirubinemia confirm biliary obstruction, and ultrasound showing a thickened, distended common bile duct further supports the diagnosis of acute suppurative cholangitis.
- Endoscopic retrograde cholangiopancreatography is the most appropriate next step, as it allows for both definitive diagnosis and urgent therapeutic decompression of the biliary system.

Predicted rationale:

This 42-year-old woman presents with right upper quadrant abdominal pain that began after dinner, which is typical of biliary colic often seen in gallbladder pathology. Her medical history of obesity and diabetes are well-known risk factors for gallstone formation, increasing the likelihood that this is a case of gallstone-induced cholecystitis. The presence of jaundice and scleral icterus can be explained by transient obstruction of the cystic duct or pressure on the common bile duct due to an inflamed gallbladder. The ultrasound showing a thickened, distended common bile duct supports the diagnosis of acute cholecystitis complicated by pericholecystic edema or secondary biliary obstruction. Her vital signs are concerning, with a fever of 104°F, tachycardia at 160/min, and hypotension at 90/65 mmHg, indicating systemic inflammatory response syndrome (SIRS) likely triggered by a gallbladder infection. The leukocytosis (22,500/mm³) further reinforces the infectious etiology. Notably, her liver enzymes (AST 42, ALT 31) are only mildly elevated, which makes primary hepatic causes less likely and supports the idea that this is primarily a gallbladder problem. While conservative management with fluids and antibiotics is essential, immediate imaging to directly assess the bile ducts is warranted. ERCP allows for visualization of the biliary tree and intervention if needed, such as sphincterotomy or stone extraction. Given the severity of her presentation and risk factors, ERCP is the most appropriate next step in management to both clarify the diagnosis and initiate definitive therapy.

Reasoning correctness: 1/3

Supplementary Figure 6. Case study of medical reasoning assessment. This case demonstrates that the prediction model Med42-70B produced a correct answer with flawed reasoning trajectories. The correct rationale is highlighted in red. It shows that LLM-w-Rationale can capture flawed reasoning patterns and offers a more nuanced evaluation of an LLM's medical capabilities.

Supplementary Note 12

We conducted an error analysis to identify cases where LLM-w-Rationale's evaluation diverged from expert assessment of medical reasoning. In the false-negative example shown in Supplementary Figure 6, LLM-w-Rationale assigned a lower score because it mistakenly believed that key information was missing. In contrast, human experts recognized that the reasoning contained the essential elements and deemed it correct.

Type: Diagnostic workup

Question: A 50 years old male presented with frontal bossing, enlarged nasal bone, enlarged jaw and spade like fingers. Which of the following test will you do for diagnosis?

Answer Choices: A. IGF1 B. ACTH C. TSH D. Serum coisol

Ground-truth answer: A. IGF1

Predicted answer: A. IGF1

Fine-grained reasoning annotation:

- Frontal bossing, prognathism (enlarged jaw), and spade-like fingers are hallmark features of acromegaly from excess growth hormone in adults.
- Insulin-like growth factor 1 (IGF-1) levels accurately reflect average growth hormone secretion and are the recommended initial diagnostic test for suspected acromegaly.

Predicted rationale:

The physical features of the patient make me think of acromegaly. But how do we test for that? The options given are IGF1, ACTH, TSH, and serum cortisol. I remember that in acromegaly, the overproduction of GH leads to increased insulin-like growth factor 1 (IGF-1). So measuring IGF-1 levels is a common initial test because it's more stable than GH levels, which can fluctuate. High IGF-1 would support the diagnosis.

LLM-w-Rationale score: 0.5

Human score: 1.0

Supplementary Figure 7. Error analysis of a false-negative case produced by LLM-w-Rationale on medical reasoning evaluation. The prediction was generated by Qwen3-32B and evaluated by the GPT-4o-mini judge model. Correct reasoning trajectories are highlighted in red, and the false-negative trajectory is highlighted in yellow.
